# Mangroves are an overlooked hotspot of insect diversity despite low plant diversity

**DOI:** 10.1186/s12915-021-01088-z

**Published:** 2021-09-14

**Authors:** Darren Yeo, Amrita Srivathsan, Jayanthi Puniamoorthy, Foo Maosheng, Patrick Grootaert, Lena Chan, Benoit Guénard, Claas Damken, Rodzay A. Wahab, Ang Yuchen, Rudolf Meier

**Affiliations:** 1 Center for Integrative Biodiversity Discovery, Leibniz Institute for Evolution and Biodiversity Science, Museum für Naturkunde, Invalidenstr. 43, Berlin, 10115 Germany; 2 Department of Biological Sciences, National University of Singapore, 14 Science 8 Drive 4, Singapore, 117543 Singapore; 3 Lee Kong Chian Natural History Museum, National University of Singapore, 2 Conservatory Drive, Singapore, 117377 Singapore; 4 National Biodiversity Centre, Royal Belgian Institute of Natural Sciences, Brussels, Belgium; 5 International Biodiversity Conservation Division, National Parks Board, 1 Cluny Road, Singapore, 259569 Singapore; 6 School of Biological Sciences, The University of Hong Kong, Kadoorie Biological Sciences Building, Pok Fu Lam Road, Hong Kong, SAR China; 7 Institute for Biodiversity and Environmental Research, Universiti Brunei Darussalam, Jalan Universiti, BE1410 Gadong, Brunei Darussalam

**Keywords:** Insect biodiversity, Mangroves, NGS barcoding, Species discovery, Beta-diversity, Global insect decline, Southeast Asia

## Abstract

**Background:**

The world’s fast disappearing mangrove forests have low plant diversity and are often assumed to also have a species-poor insect fauna. We here compare the tropical arthropod fauna across a freshwater swamp and six different forest types (rain-, swamp, dry-coastal, urban, freshwater swamp, mangroves) based on 140,000 barcoded specimens belonging to *ca.* 8500 species.

**Results:**

We find that the globally imperiled habitat “mangroves” is an overlooked hotspot for insect diversity. Our study reveals a species-rich mangrove insect fauna (>3000 species in Singapore alone) that is distinct (>50% of species are mangrove-specific) and has high species turnover across Southeast and East Asia. For most habitats, plant diversity is a good predictor of insect diversity, but mangroves are an exception and compensate for a comparatively low number of phytophagous and fungivorous insect species by supporting an unusually rich community of predators whose larvae feed in the productive mudflats. For the remaining tropical habitats, the insect communities have diversity patterns that are largely congruent across guilds.

**Conclusions:**

The discovery of such a sizeable and distinct insect fauna in a globally threatened habitat underlines how little is known about global insect biodiversity. We here show how such knowledge gaps can be closed quickly with new cost-effective NGS barcoding techniques.

**Supplementary Information:**

The online version contains supplementary material available at 10.1186/s12915-021-01088-z.

## Background

Insects are currently experiencing anthropogenic biodiversity meltdowns with declines having attracted much attention [1–4] and controversy [5–10]. The controversy is largely due to the paucity of high-quality, quantitative arthropod data with sufficient taxonomic resolution. The same paucity is also responsible for imprecise estimates of global animal species richness [11, 12] and our poor understanding of geographic and temporal species turnovers for invertebrates [13–15]. These knowledge gaps are likely to threaten the health of whole ecosystems given that arthropods provide important ecosystem services [3, 16–19], contribute much of the terrestrial animal biomass (~10%) [20], and are yet frequently ignored in habitat assessments. The lack of baseline data is particularly worrisome at a time when tropical ecosystems are heavily impacted by habitat conversion and global change [21].

The situation is particularly dire for the species-rich tropics, for which few comprehensive surveys have been conducted [22–24]. For example, only three of 73 studies in a recent review of insect declines involved tropical sites [8]. Furthermore, tropical insect surveys have traditionally focused on rainforests [24], with other habitats being largely neglected. Mangrove forests are a prime example of a tropical habitat for which the insect fauna is poorly characterized. Mangroves used to cover more than 200,000 km^2^ of the global coastline [25], but have been experiencing an annual area loss of 1–2% [25, 26]. Indeed, the losses of mangroves far exceed those of more high-profile ecosystems such as rainforests and coral reefs [26]. Unfortunately, these losses are further exacerbated by climate change [27], with some simulations predicting a further reduction by 46–59% for all global coastal wetlands by the year 2100 [28]. This is particularly worrying as mangrove ecosystems sequestrate a particularly large amount of carbon per hectare [29]. These changes will not only endanger entire ecosystems that provide essential ecosystem services [30–32], but also threaten the survival of numerous mangrove species with unique adaptations. Mangrove specialists with such adaptations are well known for vertebrates and vascular plants [33, 34], but the invertebrate diversity is poorly known.

One reason why the mangrove insect fauna may have received little attention is low plant diversity. Tropical arthropod diversity is usually strongly positively correlated with plant diversity [23, 24, 35] which suggested that mangroves would not be species-rich for insects and would thus provide few insights into one of the key questions in insect biodiversity, viz. understanding whether insect herbivores drive high plant diversity in the tropics [36–38] or vice versa [22, 39]. Arguably, the traditional focus on this question may have had the undesirable side-effect that the insect fauna of habitats with low plant diversity have received little attention. For example, the few existing studies of mangrove insects focused on specific taxa [40–42], only identified specimens to higher taxonomic levels [43–45], and/or lacked quantitative comparison with the insect fauna of adjacent habitats. Given these shortcomings, it may not surprise that these studies yielded conflicting results [44, 46, 47] with some arguing that high salinity and/or low plant diversity [33, 44, 46] are responsible for a comparatively poor insect fauna, while others reporting high levels of species diversity and specialization [47].

Here, we present the results of a comprehensive study of species richness and turnover of arthropods across multiple tropical habitats. The assessment is based on >140,000 barcoded specimens obtained over >4 years from mangroves, rainforests, swamp forests, disturbed secondary urban forests, dry coastal forests, and freshwater swamps in Singapore (Additional file 2: Figure S1). In addition, we assess the species richness and turnover of mangrove insects across East and Southeast Asia by including samples from Brunei, Thailand, and Hong Kong. Specifically, our study (1) estimates mangrove insect diversity, (2) evaluates the distinctness in reference to five different forest habitats, (3) analyzes the biodiversity patterns by ecological guild, and (4) determines species turnover across Southeast and East Asia. Most of the work was carried out in Singapore because it has a large variety of different habitats that occur within 40 km on a small island (724 km^2^) that lacks major physical barriers. In addition, all habitats have experienced similar levels of habitat degradation or loss (>95% overall loss of original vegetation cover [48], *ca.* 90% loss of rainforest [49], *ca.* 93% loss of swamp forest [50], 91% loss for mangroves [51]).

A thorough assessment of insect biodiversity requires dense sampling over an extended period of time [52–54]. We sampled 107 sites in Singapore using Malaise traps. All samples were subsequently sorted to 13 arthropod orders (Additional file 1: Table S1) with Diptera and Hymenoptera contributing the largest proportions of specimens (>75%: Additional file 1: Table S1, see Additional file 2: Figure S2 for species composition). We here analyze the diversity patterns for a subset of taxa that represent different ecological guilds (see the “Materials and Methods” section; Additional file 1: Table S2). The NGS-barcoded 140,000 specimens [55] were grouped into putative species. This allowed for estimating species richness and abundance [56–58]. Contrary to expectations, we find that mangrove forests have a very distinct and rich insect fauna. In addition, the species turnover for all habitats in Singapore and the different mangrove sites in Asia is high.

## Results

### Species delimitation based on NGS barcodes

We initially obtained 144,865 313-bp *cox1* barcodes but then excluded from analysis those Malaise trap samples that contained <100 specimens for the target taxa. The remaining 144,356 specimens were clustered into 8256–8903 molecular operationally taxonomic units (mOTUs, henceforth referred to as species) using objective clustering [59] at different p-distance thresholds in order to assess the sensitivity of results to clustering thresholds (2–4%; Additional file 1: Table S3). A further assessment of robustness was based on using an alternative species delimitation algorithm, USEARCH [60], which yielded similar species richness estimates of 8520–9315 species using the identity (--id) parameters 0.96–0.98. Most species boundaries were stable, with species numbers only varying by <12% across species delimitation techniques and parameters. We therefore used the species generated via objective clustering at 3% p-distance for the analyses, but Additional file 2: Figure S3 presents the results obtained for 2% and 4%. We initially analyzed a core dataset consisting of 62,066 Diptera and Hymenoptera specimens (4002 species at 3% p-distance; see Additional file 1: Table S1). Thereafter, we broadened the analysis to the full dataset (144,356, see the Methods section for sampling details) including data for all orders and sites. We found that the conclusions for mangrove insect diversity were consistent across the datasets. Therefore, subsequent analyses were based on the full dataset, but the corresponding results for the core dataset are presented in Additional file 2: Figures S4 and S5.

### Alpha-diversity across habitats

**Fig. 1:**
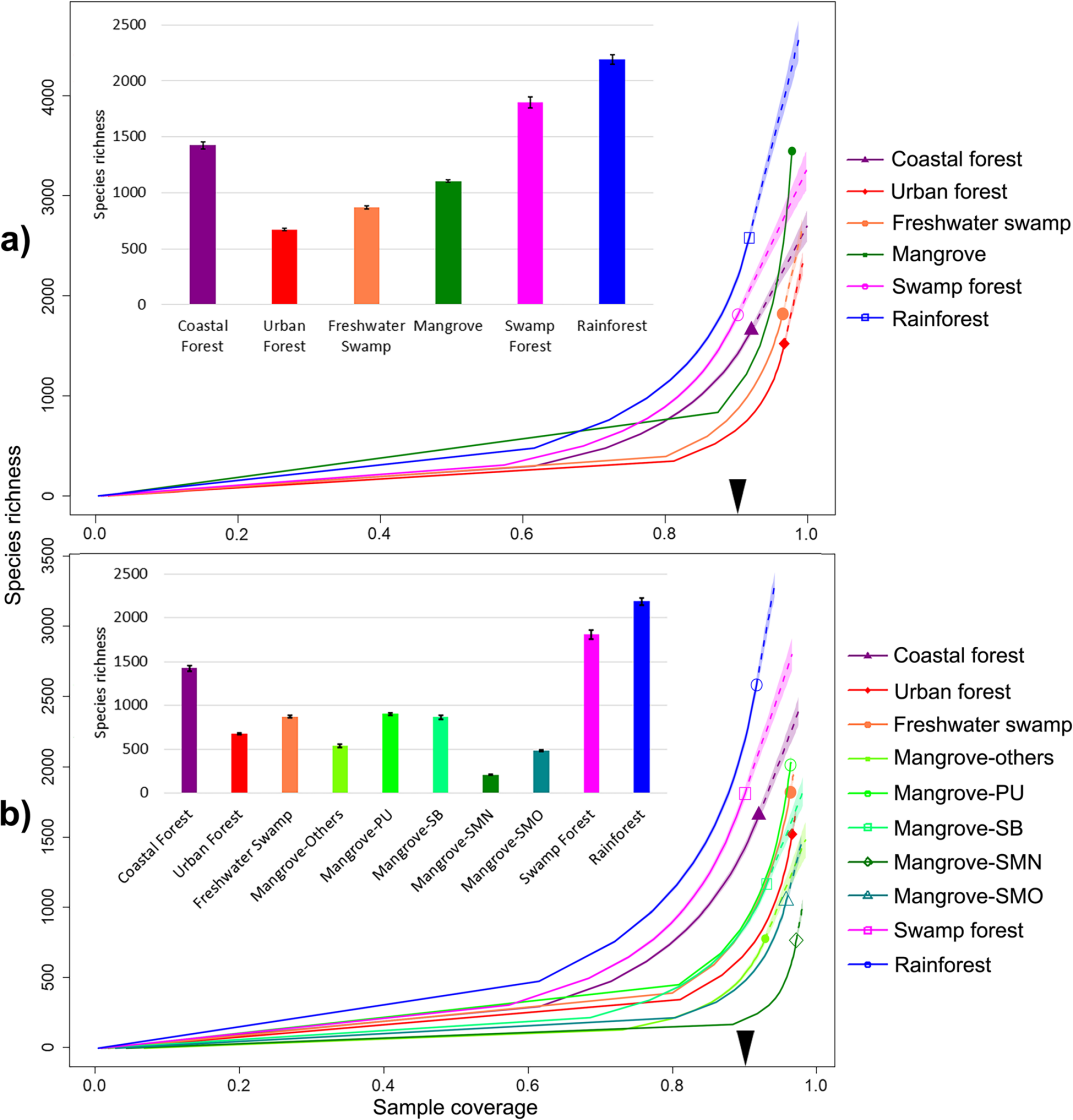
Insect alpha-diversity across tropical forest habitats. **a** Mangroves treated as one habitat; **b** Comparison of mangrove sites: Pulau Ubin (PU), Sungei Buloh (SB), Pulau Semakau old-growth (SMO), Pulau Semakau new-growth (SMN), other smaller mangrove fragments (see Additional File 1: Table S13); solid lines = rarefaction; dotted = extrapolations. The arrow on the x-axis indicates the point of rarefaction where species richness comparisons were made (see bar charts for absolute numbers with 95% confidence intervals)

We rarefied the species richness curves by sample coverage [61] (Fig. 1) for each habitat, as well as by the number of barcoded specimens (Additional file 2: Figure S3). Alpha-diversity comparisons were made at the rarefaction point with the lowest coverage/number of specimens (i.e., swamp forest in Fig. 1a). The initial analysis treated mangroves as a single habitat type. The species diversity of mangroves (1102.5 ± 10.8 species) was ca. 50–60% (core dataset: 40–50%) of the rarefied species richness of adjacent tropical primary/secondary forest (2188.4 ± 42.6 species) and swamp forest sites (1809 species) (Fig. 1a). A separate analysis of two major mangrove sites (PU & SB) revealed that they have similar species richness as the freshwater swamp site after rarefaction (Fig. 1b). The species richness of a third mangrove site (SMO) was lower and more similar to the richness of an urban forest site. A newly regenerated mangrove (SMN), adjacent to an old-growth mangrove (SMO) had much lower species richness.

We also attempted to estimate species richness using extrapolation via frequency ratio [62] and combined non-parametric estimators (CNE) [63], with the former being widely used to estimate microbial diversity [64, 65]. Both estimators suggest that mangroves are even more species-rich than rainforest and swamp forest habitats (Additional file 2: Figure S4) with coastal- and urban forests yielding even higher estimates. However, the confidence intervals were very large and the observed species richness was only 21–90% of the estimated richness which indicated that our data remain insufficient for comparing species richness based on extrapolation [66].

### Species turnover across habitats

**Fig. 2:**
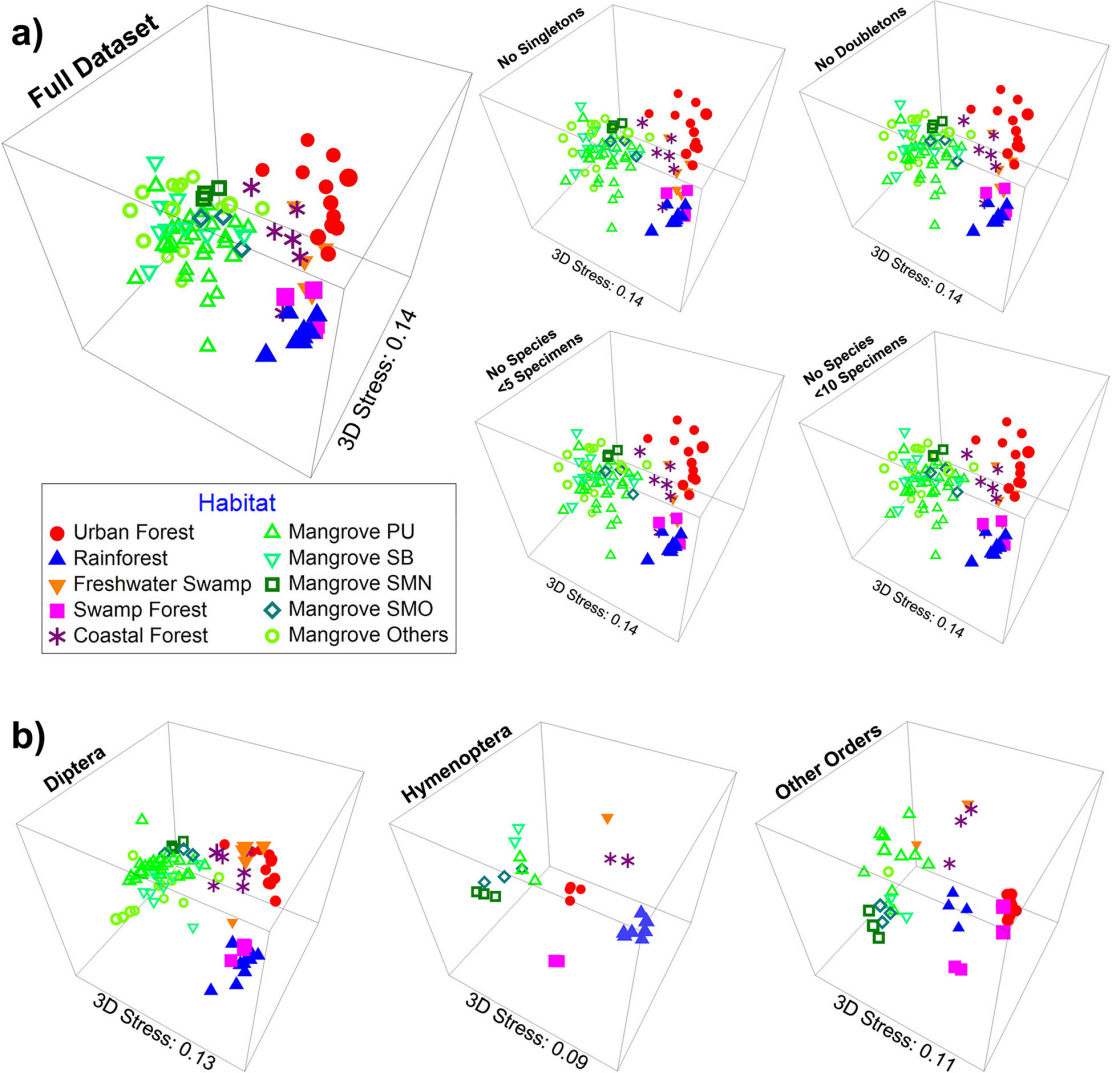
Insect communities across tropical forest habitats are distinct based on Bray-Curtis distances illustrated on 3D NMDS plots. Results are stable even when rare species are removed (**a**) or the data are split into different taxonomic groups (**b**)

Arthropod communities from most habitats are well separated on NMDS plots. This includes the mangrove insect fauna (Fig. 2) although the geographic distances between some mangrove sites (PU, SB, SM) are higher (>30 km) than the distances to other habitat types (Additional file 2: Figure S1). This separation by habitat is also evident in the core dataset (Additional file 2: Figure S6), and the same patterns are observed when the data are split into three taxa: (1) Diptera, (2) Hymenoptera, and (3) remaining arthropods (Fig. 2b). This habitat-driven structure was verified with a multivariate analysis of variance (MANOVA) test on the NMDS1 & NMDS2 coordinates, with habitat being a highly significant variable (*p* < 0.001) in explaining the variance in the combined NMDS coordinate distributions and each coordinate separately. These results are also robust to the removal of rare species (Fig. 2a; applies to NMDS plots and MANOVA). Only 48 (0.6%) of the 8572 putative species in the species turnover analysis are found in all habitat types while 5989 (69.9%) are only found in a single one (Additional file 1: Table S4); 3557 species are only known from mangroves which shared the largest number of species with coastal forests (873 of 3557 species). When rare species are removed (<10 specimens), 481 of the remaining 1773 species (27.1%) are found in a single habitat while only 48 (2.7%) are found in all (Additional file 1: Table S4); i.e., even after excluding rare species, a large proportion of the insect communities are putative habitat specialists.

The dissimilarity of the habitat-specific communities was confirmed via tests with *mvabund* [67]*,* which finds that the variable “habitat” is highly significant (*p* < 0.001) in determining community structure, even when rare species were excluded. This differentiation is further supported by ANOSIM tests (Table 1), which find significant differences between communities in both global (*P* = 0.001, R = 0.784) and pairwise habitat comparisons (*P* = 0.001–0.019, R = 0.341–0.983). The only exceptions are the coastal and urban forests (*P* = 0.079, R = 0.172) which may be due to the close proximity of Pulau Ubin coastal forest sites to urban settlements (Additional file 2: Figure S1). Note that a SIMPER analysis (Table 2) finds a substantial number of shared species between the rainforest and swamp forest sites (13.88%). Both sites are geographically close (<5km; Additional file 2: Figure S1) and the within-habitat values for both sites are fairly high (rainforest = 29.59%, swamp forest = 31.10%). ANOSIM and SIMPER results are again robust to the removal of rare species (Additional file 1: Tables S5 & S6), and the ANOSIM *p* values for most comparisons are significant even according to re-defined statistical criteria for unexpected or new results (*p* < 0.005) [68]. The observed dissimilarity was largely due to species turnover given that the turnover component (0.898) greatly exceeded nestedness (0.048), which can also generate high dissimilarity between communities if one is a small subset of the other (Table 3 & Additional file 1: Table S7). This was similarly observed in most pairwise comparisons of habitats (turnover = 0.704–0.956, nestedness = 0.001–0.102). The only exception was mangroves and coastal forests (turnover = 0.658, nestedness = 0.254) which are in close geographic proximity on Pulau Ubin (Fig. 1).

**Table 1:** Species turnover across habitats. Distinctness of communities in each habitat type as assessed with ANOSIM (pairwise *p* value below and R-statistics above diagonal)

Overall P: 0.001			Overall R: 0.784			
	Rainforest	Urban forest	Swamp forest	Mangrove	Freshwater swamp	Coastal forest
**Rainforest**		0.817	0.983	0.953	0.973	0.955
**Urban forest**	0.001		0.759	0.815	0.575	0.172
**Swamp forest**	0.001	0.001		0.934	0.769	0.893
**Mangrove**	0.001	0.001	0.001		0.856	0.546
**Freshwater swamp**	0.001	0.001	0.008	0.001		0.341
**Coastal forest**	0.001	0.079	0.005	0.001	0.017

**Table 2:** Species turnover across habitats. Distinctness of communities in each habitat type as assessed with SIMPER

	Within habitat (%)	Between habitats (%)					
		Rainforest	Urban forest	Swamp forest	Mangrove	Fresh-water swamp	Coastal forest
**Rainforest**	29.59						
**Urban forest**	12.91	3.20					
**Swamp forest**	31.10	13.88	2.94				
**Mangrove**	12.25	1.62	3.09	1.98			
**Freshwater swamp**	17.29	2.13	4.69	4.10	2.74		
**Coastal forest**	12.09	3.82	9.41	4.00	6.08	9.05

**Table 3:** Species turnover across habitats. Species turnover and nestedness analysis (pairwise turnover values below and nestedness above diagonal)

Overall Dissimilarity: 0.946		Overall Turnover: 0.898			Overall Nestedness: 0.048	
	Rainforest	Urban forest	Swamp forest	Mangrove	Freshwater swamp	Coastal forest
**Rainforest**		0.011	0.072	0.054	0.007	0.021
**Urban forest**	0.916		0.028	0.097	0.005	0.102
**Swamp forest**	0.710	0.922		0.092	0.027	0.001
**Mangrove**	0.914	0.819	0.878		0.062	0.254
**Freshwater swamp**	0.956	0.891	0.932	0.878		0.093
**Coastal forest**	0.908	0.704	0.940	0.658	0.756	

### Relationship between insect and plant richness

Compared to mangroves (ca. 250 plant species), the rainforest and swamp forest sites have 4.6 or 7.6 times the number of recorded species based on checklists for the sites (Additional file 1: Table S8). This higher species richness is also confirmed by plot data for the rainforest [69] (839 species in 52 plots of 100m^2^) and swamp forest [70] (671 species in 40 plots of 400m^2^). However, the insect biodiversity of the rainforest and swamp forest sites is only 1.64–1.98 times higher than in the mangroves after rarefaction (1.99–2.52 times higher in the core dataset).

### Analysis of ecological guilds and correlation between insect and plant diversity

**Fig. 3:**
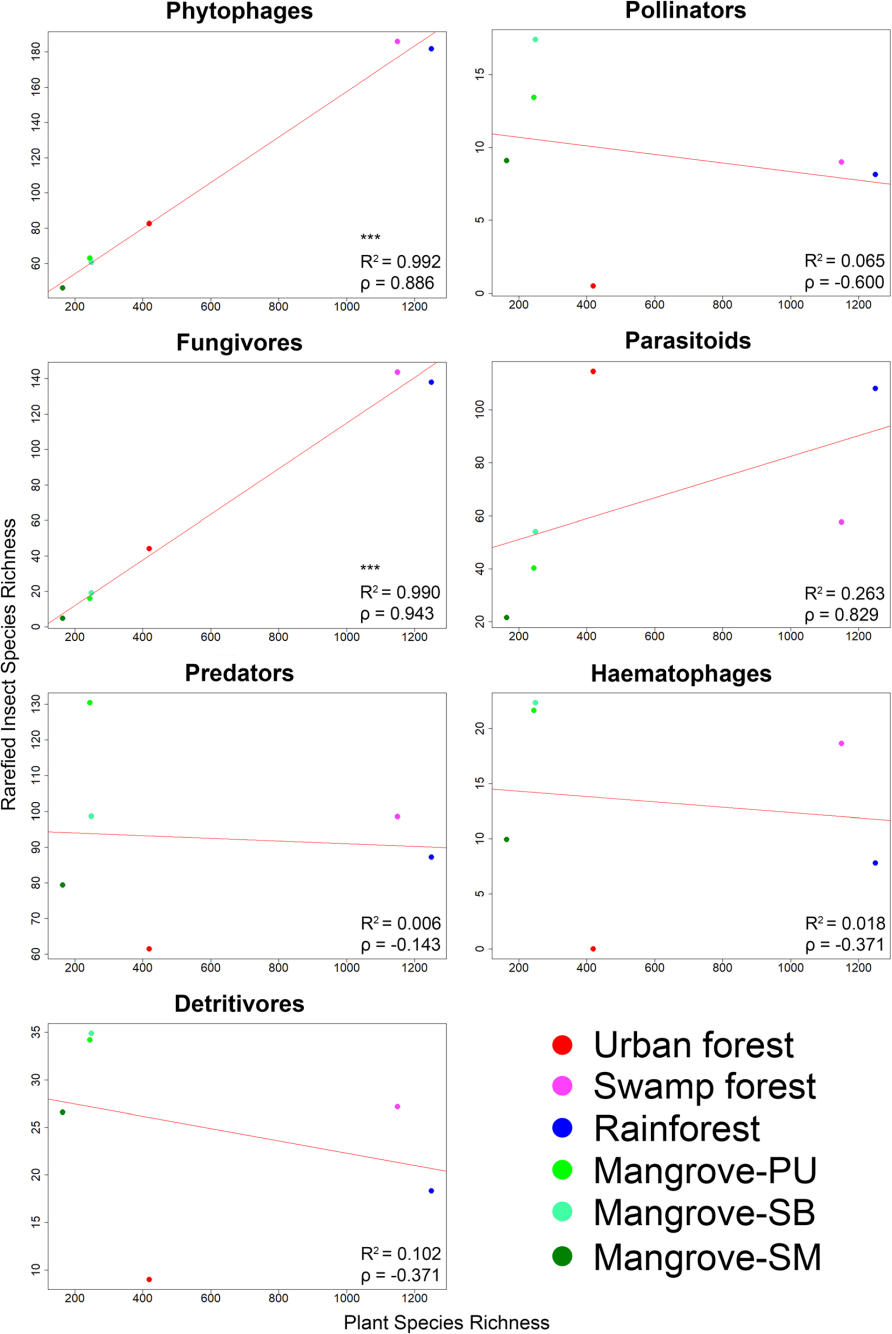
Only the diversity of phytophagous and fungivorous insects is correlated with plant diversity based on a linear regression model using rarefied insect species richness (*≤0.05, **≤0.01, ***≤0.001). Color coding of points as in Fig. 2

For this analysis, we used the core dataset which was obtained from traps with at least 6 months continuous sampling covering a dry- and wet season (April–September). We only analyzed some Diptera and Hymenoptera taxa that were sufficiently abundant and could be assigned to a broad range of ecological guilds. Note that these orders were chosen because they dominate Malaise trap samples (see Brown 2005 [71] & Hebert et al. 2016 [72]). To understand how different habitats maintain insect diversity, we assigned insect species with known family/genus identities to ecological guilds (42,092 specimens belonging to 2230 putative species; no guild information is available for the remaining 19,974 specimens). After stepwise refinement of an analysis of covariance (ANCOVA) model, the final model was defined as: *insectdiv ~ habitat + guild + plantdiv + guild:plantdiv* (*insectdiv*: insect species richness, *plantdiv*: plant species richness). The type-II sum of squares test reveals that guild and the interaction term between guild and plant diversity are highly significant (*p* < 0.001), while plant diversity (*p* = 0.063) and habitat (*p* = 0.468) are not. This suggests guild and plant diversity together play an important role in determining insect diversity but the precise relationship warranted further testing. Single variable linear regressions (*insectdiv ~ plantdiv)* were performed on each guild separately (Fig. 3), and only plant diversity was found to be highly significantly and positively correlated with the alpha-diversity of phytophagous and fungivorous insects (*p* < 0.001, R^2^ = 0.992 and 0.990, *p* = 0.886 and 0.943, respectively).

**Fig. 4:**
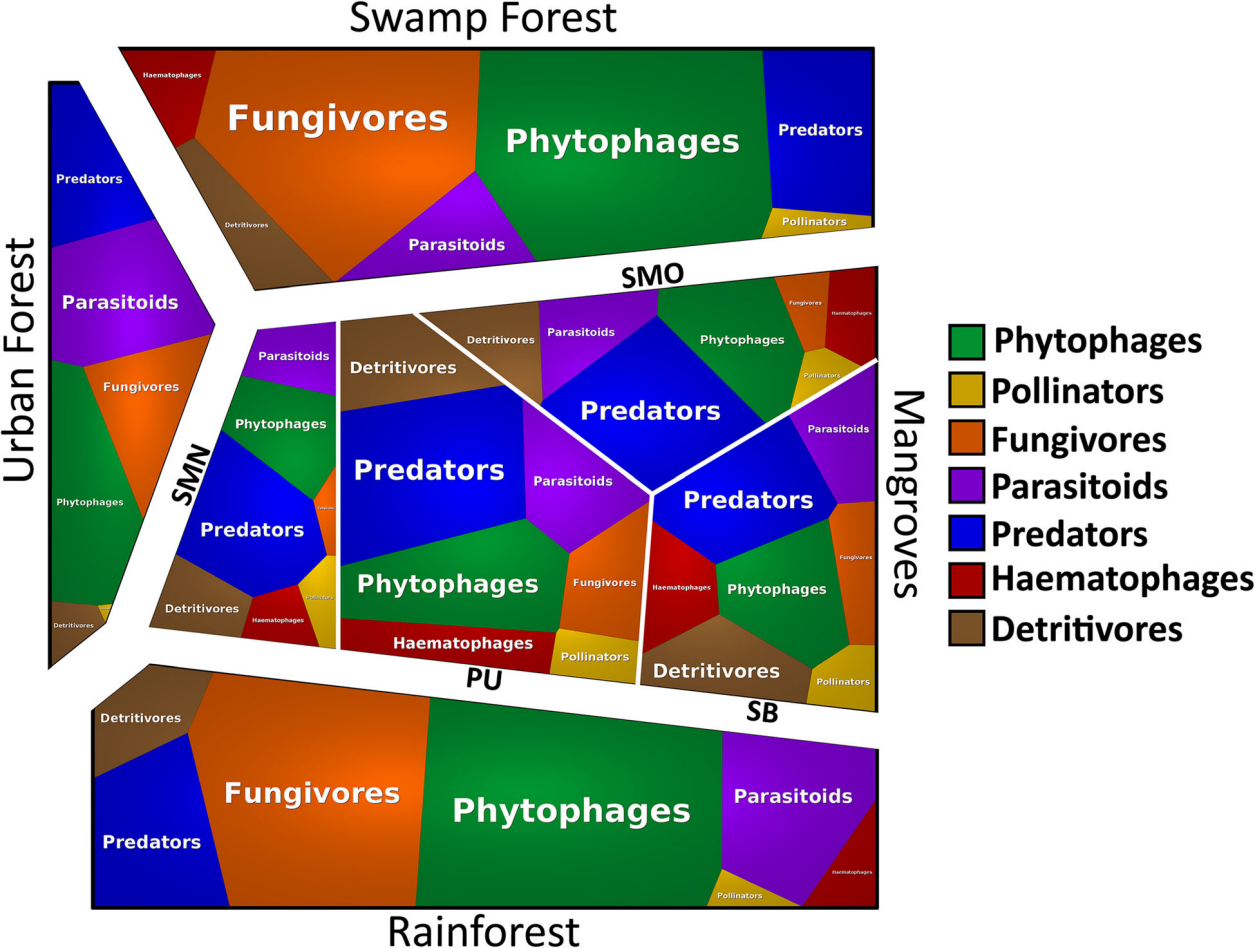
Voronoi treemap of insect guilds across four habitats. Mangroves are represented by four sites (PU=Pulau Ubin, SB=Sungei Buloh, SMO: Semakau old mangrove, SMN: Semakau restored mangrove). Phytophages and fungivores dominate the rain and swamp forests while predators are overrepresented in mangroves

**Fig. 5:**
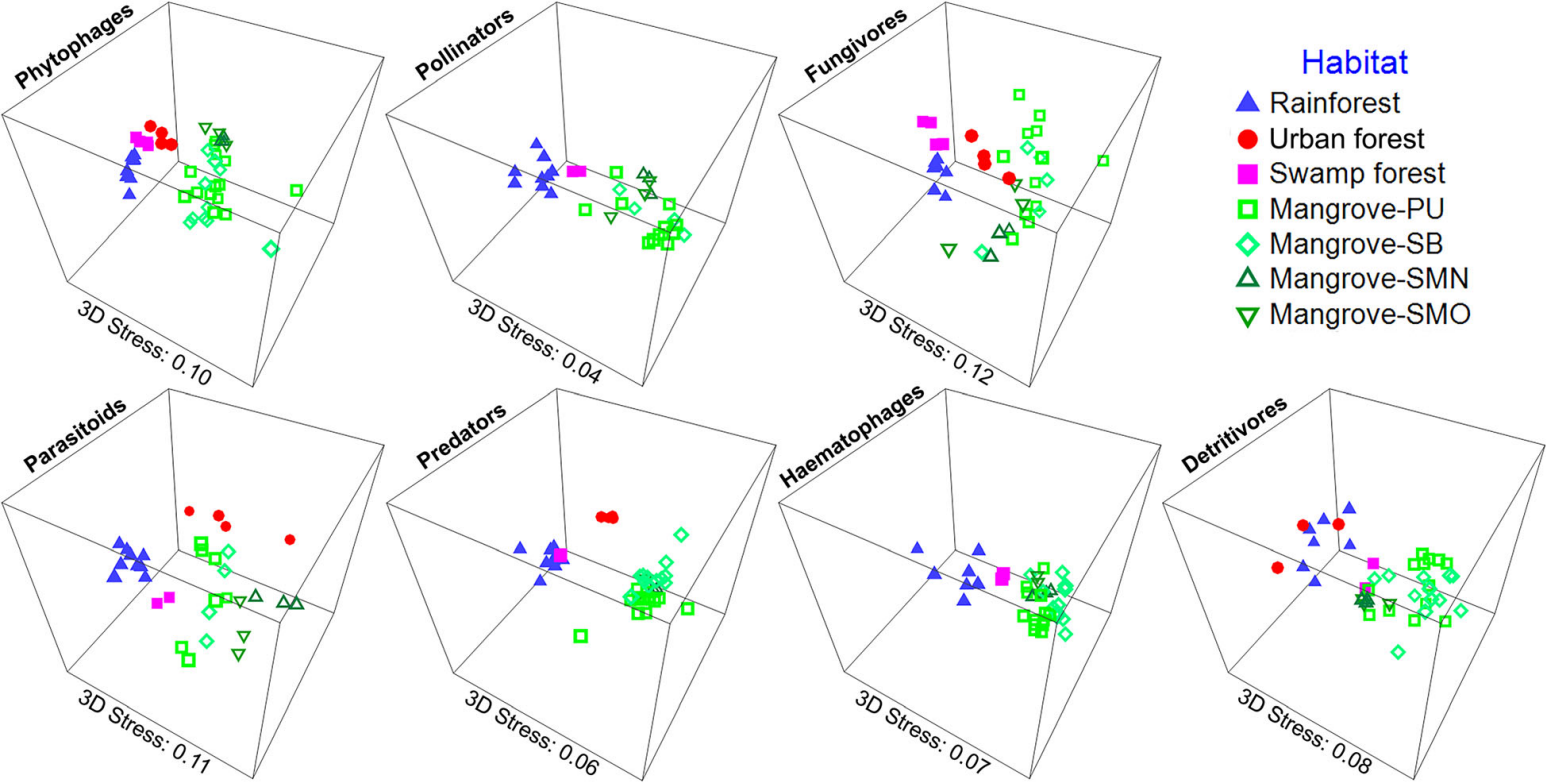
Habitat differentiation by insect guilds (3D NMDS plot of Bray-Curtis distances for habitats with >2 sites)

The different habitat types vary in composition (Fig. 4 & Additional file 1: Table S9). Rainforest and freshwater swamp forest sites have higher numbers and proportions of phytophagous and fungivorous insect species (see also Additional file 2: Figures S7 & S8). The insect communities of mangroves, however, are characterized by an elevated proportion of predatory species. With regard to species turnover, insect communities are separated by habitat for most guilds and in most pairwise comparisons (Fig. 5, Additional file 1: Tables S10 & S11).

### Species turnover across Asian mangroves

The specimens from Hong Kong belonged to 109 dolichopodid, 129 phorid, and 25 mycetophilid species. The corresponding number for Brunei were 96 and 76 species for dolichopodids and phorids, with too few mycetophilids being available for evaluation (Additional file 1: Table S12). The southern Thai dolichopodids belonged to 74 species. We find high species turnover between Hong Kong, Brunei, and Singapore, even after rarefying the specimen sample sizes (Additional file 2: Figure S9). Approximately 90% of all dolichopodid and phorid species are unique to each region with <1% shared across all regions. Species turnover is even higher for the mycetophilids of Hong Kong and Singapore (>95%). Species turnover for the dolichopodids of Southern Thailand and Singapore is again high with only 11.5% of all species shared between both countries.

## Discussion

### Discovery of a largely overlooked, predator-enriched insect community in mangroves

It is often assumed that the insect diversity in mangroves is low because high salinity and low plant diversity are thought to be unfavourable to insect diversification [23, 73, 74]. However, we here show that mangroves are species-rich for insects despite low plant diversity (<250 species: [75–77]). This result is supported by rarefaction and species richness estimation with frequency ratio and CNE estimators although the latter yield highly variable estimates that triple the observed number of species [66]. In addition to being species-rich, the mangrove fauna is also very unique. More than half of its species are not found in other habitats, even though coastal forests are directly adjacent to mangroves. Indeed, after adjusting for sampling effort, the species diversity in Singapore’s premier rainforest reserve (Bukit Timah Nature Reserve: 1.64 km^2^) and largest swamp forest remnant (Nee Soon: 5 km^2^) is only 50% higher than the diversity of major mangrove sites (PU: 0.904 km^2^, SB: 1.168 km^2^, SM: 0.174 km^2^). The high diversity encountered in the mangrove sites was particularly unexpected because the rainforests of Bukit Timah Nature Reserve have been protected for more than 50 years [78, 79] and have very high plant diversity (e.g., 1250 species of vascular plants [69] including 341 species of trees [80] in a small 2 ha plot of the Centre for Tropical Forest Science). Moreover, we extensively sampled the insect diversity in this reserve by placing multiple Malaise traps in primary, maturing secondary, and old secondary forests. Similarly, we expected the insect diversity of Singapore’s largest swamp forest (Nee Soon) to greatly exceed the number of species found in the mangrove sites because this swamp forest site is known for its high species richness (e.g., 1150 species of vascular plant species [81]).

A guild-level analysis reveals that rainforests and swamp forests have overall the highest species diversity for most guilds (Additional file 2: Figure S7). Mangroves maintain high species diversity although they are impoverished for phytophagous and fungivorous species. Mangroves are, however, home to a disproportionally large proportion of predatory species (Fig. 4) whose larvae develop in sediments (Empidoidea and Tabanidae). This suggests that the high insect diversity in tropical habitats may be achieved by having large proportions of species developing in the biologically most productive microhabitats—plants and fungi for many forest habitats and the highly productive mud flats for mangroves.

In addition to finding high alpha-diversity in mangroves, we also document that the mangrove insect communities are very distinct. This conclusion is supported by a multitude of analyses (mvabund, ANOSIM, SIMPER, NMDS). It is furthermore insensitive to the removal of rare species (Fig. 2) and driven by high species turnover rather than nestedness (see Table 3). This stratification by habitats is still evident even when the two dominant insect orders in Malaise trap samples (Diptera and Hymenoptera) are removed (Fig. 2). Comparatively high overlap is only observed between mangroves and coastal forests which is presumably due to the close proximity of the habitats on Pulau Ubin where back mangroves and coastal forests are contiguous (Additional file 2: Figure S1). The uniqueness of the mangrove insect community is probably due to the unusual environmental conditions characterized by extreme daily fluctuations in salinity, temperature, and inundation. These extreme conditions are likely to require physiological and behavioral adaptations that support an evolutionarily distinct fauna. What is surprising, however, is that we find no evidence for an adaptive radiation of particular clades. Instead, a large number of independent colonization events seems more likely given that the mangrove species usually belong to genera that are also known from other habitats (e.g., Dolichopodidae). This challenges the view that high salinity is a potent colonization barrier for invertebrates [73, 74] over evolutionary time scales.

Mangrove regeneration is pursued in many countries, with mixed successes in restoring the original plant diversity [82, 83], but it remains poorly understood whether regenerated mangroves harbour the original arthropod biodiversity. Our preliminary data based on 311 Malaise trap samples from one regenerated site suggests that this is not the case. The regenerated mangrove (SMN) was replanted with a monoculture of *Rhizophora stylosa* [77] which replaced old-growth mangroves that had been cleared during reclamation (1994–1999 [51]). The restored site (SMN) has markedly lower insect species richness than all other mangrove sites, including a neighbouring old-growth mangrove (SMO; Fig. 1). This highlights once more that habitat assessments have to be holistic and should not only be based on plant and vertebrate data [84].

Mangrove insect communities are not only rich and distinct in Singapore. Within Asia, we reveal a 92% species turnover between Singapore and Hong Kong (2,500 km north; Additional file 2: Figure S1) for taxa representing different guilds (Dolichopodidae–predators: 483 species, Mycetophilidae–fungivores: 67 species, Phoridae–mostly saprophagous: 591 species). While climatic differences could be advanced as a potential explanation, comparisons with the mangroves in the geographically close and tropical Borneo (Brunei) confirm a high species turnover of 85% (see also Grootaert 2019 [85]). Further evidence for high regional species turnover in mangroves emerges when the dolichopodid fauna of Singapore’s and Brunei’s mangroves are compared with the fauna of Southern Thailand (coasts of South China and Andaman seas). Only 34 and 10 of the 74 known Thai species are shared with Singapore and Brunei, respectively. These data suggest that a significant proportion of the global insect diversity may reside in mangroves. Based on the data from Singapore, it appears that much of the diversity may be resilient to disturbance, given that we find no evidence that the insect diversity in Singapore’s mangrove fragments is depressed relative to what is found in the much more pristine sites in Brunei. This also suggests that the loss of species diversity for small, flying insects in Singapore may not have been as dramatic as what has been observed for Singapore’s vertebrate and large invertebrate species [48, 86, 87].

### Discovering a new insect hotspot with NGS barcoding

Global insect declines have recently received much attention by the scientific community [2] and public [88]. Obtaining relevant data is very slow and expensive when conventional techniques are used. This is so because too many specimens have to be sorted into too many species before a holistic habitat assessment can be carried out [89]. In our study, this problem is overcome by specimen sorting using NGS barcodes, which differ from traditional barcodes by costing only a fraction of those obtained with Sanger sequencing. Yet, species richness estimates based on 313-bp NGS barcodes have a 90% congruence with estimates based on morphological data [56, 57, 90, 91]. This suggests that large-scale species discovery with NGS barcodes can yield sufficiently accurate information on species abundance and distribution for habitat assessments [55, 56]. This also means that NGS barcodes could be used for quickly revealing hidden hotspots of insect diversity in countries with high diversity and limited science funding. We estimate that the ~140,000 specimens in our study could today be obtained for <USD25,000 using 350 manpower days whereas a similar study based on morphology would require >150 manpower years [92]; i.e., some of the traditional obstacles to understanding arthropod biodiversity caused by the taxonomic impediments may be finally disappearing. However, it is important to remember that barcode-derived units are only proxies for formal species and should only be used for broad analyses of diversity patterns. Additional taxonomic work may uncover, for example, rapid radiations in particular habitats or guilds that would be overlooked by studies that rely on clustering barcodes based on similarity only [93]. This is why the rich dolichopodid fauna is currently being revised using the “reverse workflow” approach where morphological validation follows pre-sorting with barcodes [56, 85, 94].

## Conclusions

We here document that the insect fauna inhabiting mangroves is not only rich, but also distinct. The discovery of such an unexpectedly rich and distinct insect community highlights how little we know about global arthropod diversity. Accelerating species discovery for arthropods is a pressing task given that many undersampled habitats are disappearing at a much faster rate than tropical rainforests and coral reefs. Fortunately, advances in sequencing technology will facilitate this work and we predict that mangroves will be only one of many additions to the fast growing list of habitats that have only recently been recognized as biodiversity hotspots (e.g., dry forests [95, 96], forest savannahs [97, 98]).

## Methods

### Sampling site, sample collection, and processing

Singapore has a large number of tropical habitat types that are all within 40 km of each other without being separated by major physical barriers. This allowed us to sample rainforests (from early secondary to mature secondary forest), urban-edge forests, mangroves, swamp forests, freshwater swamps, and dry coastal forests. Note that these habitats have in Singapore experienced similar levels of habitat degradation or loss due to urbanization (>95% loss of original vegetation cover [48], *ca.* 90% loss for rainforests [49], *ca.* 93% loss of swamp forest [50], 91% loss for mangroves [51]). The freshwater swamp differs from the swamp forest by lacking mature trees, while the dry coastal forests are distinct from the mangroves by lacking typical mangrove tree species. We sampled these habitat types using 107 trapping sites (Additional file 2: Figure S1). The mangrove sites were located primarily along the North-western and Southern coasts of the mainland, as well as on offshore islands in the south and northeast. The major mangrove sites were on Pulau Ubin (PU), Sungei Buloh (SB), and Pulau Semakau (SM), and the last of which is represented by an old-growth (SMO) and a newly regenerated mangrove fragment (SMN). Other smaller mangrove fragments across the island were also sampled (Mandai Nature Park, Pulau Tekong, Sarimbun, Labrador Park, Lim Chu Kang, Coney Island—see Additional file 1: Table S13) and were labeled “Mangrove—others” in the diversity analyses. The swamp forest site (Nee Soon) was Singapore’s largest remaining freshwater swamp remnant which is known for a rich insect fauna [99], overall high species richness, and level of endemism [100, 101]. Bukit Timah Nature Reserve was selected as the tropical rainforest site given its high species diversity and protected status [78]. This reserve consists of forests in various stages of succession, and hence, we sampled different forest types with three sites each being in primary forest, old secondary forest, and maturing secondary forest. The “urban secondary forest” sites were located along a disturbance gradient ranging from the campus of the National University of Singapore (NUS) through several urban parks and forest edges in Central and South Singapore. The freshwater swamp site is located primarily in Kranji, a freshwater marsh at the flooded edge of a reservoir. The “coastal forest” sites were dry secondary forests adjacent to the coast at Labrador Park and Pulau Ubin.

All specimens were collected between 2012 and 2019 (Additional file 1: Table S13) using Malaise traps. These traps are widely used for insect surveys because they are effective sampling tools for flying insects and allow for standardized, long-term sampling. Note that the use of Malaise traps in our study was appropriate because the canopy height was comparable for most habitats given that we compared mature mangroves (PU, SB, and SMO) with a wet swamp forest site, and different kinds of secondary forests. Only the canopy height of some sites in Bukit Timah Nature Reserve (BTNR) was higher (pers. obs.), but for BTNR, we also included secondary forests and several traps were placed on steep slopes that would be able to sample canopy-active fauna from a lower elevation. With regard to the habitat patches, the fragments were larger for the rainforest and swamp forest than for any of the mangrove sites (tropical rainforest: 1.64 km^2^; swamp forest: 5 km^2^, mangrove forest fragments: 0.904 km^2^ [PU], 1.168 km^2^ [SB], and 0.174 km^2^ [SM] [51]). Malaise traps in the mangroves were set up in the intertidal zone. Each Malaise trap sample consisted of 1-week’s worth of insects preserved in molecular grade ethanol.

After an ethanol change, the specimens were sorted by para-taxonomists (Additional file 1: Table S1) into mostly orders or families: Arachnida, Blattodea: cockroaches, termites, Coleoptera: Cicindelinae, Mordellidae, Staphylinidae, Elateridae, Cantharidae, Buprestidae, Curculionoidea, other Coleoptera, Diptera: Tipuloidea, Mycetophilidae, Keroplatidae, Culicidae, Chironomidae, Sciaridae, other Nematocera, Dolichopodidae, other Empidoidea, Stratiomyidae, Syrphidae, Asilidae, Tabanidae, Phoridae, other acalyptrates, Calyptrates, Dermaptera, Hemiptera: Reduviidae, Dipsocoromorpha, other Heteroptera, Auchenorrhyncha, Sternorrhyncha, other Homoptera, Hymenoptera: Apoidea, Formicidae, Lepidoptera, Mantodea, Neuroptera, Orthoptera, Psocodea, and Trichoptera. In some cases, we used broader categories that could be communicated to the parataxonomists but did not represent formal taxa (“other acalyptrates”). All subsequent barcoding used these subsamples as sampling units. Overall, the samples were typical for Malaise traps in that they were dominated by Diptera and Hymenoptera (>75% of specimens: Additional file 1: Table S1, see Additional file 2: Figure S2 for species composition). We therefore first developed a core dataset for these orders given that the specimen numbers were sufficiently high (62,066 specimens, 4002 species at 3% p-distance). This core dataset consisted of 12 Diptera families, “other Brachycera”, ants (Formicidae), and Apoidea (see Additional file 1: Table S1). This dataset also only included specimens from sites that were sampled from 2012 to 2015 and overlapped with regard to the April to September period which covers a dry and wet season. We subsequently added the available data for other sites and orders. For most of these, we barcoded all available specimens (Araneae, Blattodea, Dermaptera, Mantodea, Neuroptera, Orthoptera, Trichoptera). Three insect orders had intermediate abundances (Coleoptera, Lepidoptera, Hemiptera, Psocodea). For Coleoptera, we processed easily recognized families by barcoding all specimens collected before 2019 (Staphylinidae, Cleridae, Cerambycidae, Scirtidae, Carabidae, Elateridae). For Lepidoptera, we processed all large specimens and only subsampled micromoths. For Hemiptera and Psocodea, we sequenced all Heteroptera, but only subsampled the psocodean and homopteran planthoppers because the abundances were too high (planthoppers: 6987 specimens across 6 habitats; Psocodea: 539 specimens across 4 habitats). Here, the subsampling was similar to what had been done for the core data set in that we barcoded all specimens for the samples of particular time periods.

After obtaining barcodes, the taxa in the core dataset were re-identified to genus or family based on DNA barcodes that were submitted to the Global Biodiversity Information Facility (GBIF: www.gbif.org) or the Barcode of Life Data (BOLD: www.boldsystems.org) databases or through morphological re-examination if the identifications were ambiguous. For the former, we only used matches above 95% and 97% similarity for family- and genus-level matches, respectively. In some cases, these identifications revealed sorting errors by the parataxonomists. The barcodes were nevertheless kept for the diversity analyses because such sorting errors are random across samples.

The mangrove specimens from Hong Kong were collected by 24 Malaise traps installed between October 2017 to October 2018, while those from Brunei were collected by six Malaise traps from July to November 2014. Dolichopodidae, Phoridae, and Mycetophilidae were pre-sorted and send for barcoding to Singapore. Note that the mangrove forests in Brunei are less affected by urbanization than those in Singapore. The dolichopodid specimens from Thailand were obtained by different techniques including sweep-netting from 42 mangrove sites over a period of 15 months from Mar 2014 to Dec 2015.

### Putative species sorting with NGS barcoding

NGS barcoding combines the advantages of cost-effective sequencing via Illumina with the approximate species-level resolution provided by DNA barcodes. The molecular procedures can be learned in hours, and several hundred specimens can be processed per person and day. The overall barcode costs are now <10 cents per specimen if Illumina Novaseq is used for sequencing (2 cents/barcode based on USD 6900 per 250-bp PE flow cell yielding 800 million reads: https://research.ncsu.edu/gsl/pricing). We used NGS barcoding to amplify and sequence a 313-bp fragment of the cytochrome oxidase I gene (*cox1*) using a protocol described in Meier et al. [55]. Direct-PCR [102] was conducted for specimens collected early in the study; during this phase, we used 1–2 legs of the specimen as template for obtaining the amplicon with the primer pair mlCO1intF: 5′-GGWACWGGWTGAACWGTWTAYCCYCC-3′ [103] and jgHCO2198: 5′-TANACYTCNGGRTGNCCRAARAAYCA-3′ [104]. For samples processed later, the whole specimen was immersed in Lucigen QuickExtract solution or HotSHOT buffer [105] and gDNA extraction was conducted non-destructively. The gDNA extract was then used as a PCR template with the aforementioned reagents and protocol. The primers used were labelled with 9-bp long barcodes that differed by at least three base pairs. Every specimen in each sequencing library was assigned a unique combination of labelled forward and reverse primers, which allowed the Illumina reads to be binned according to specimen. A negative control was prepared and sequenced for each 96-well PCR plate. Amplification success rates for each plate were assessed via gel electrophoresis for eight random wells per plate.

The amplicons were pooled at equal volumes within each plate and later pooled across plates. Equimolarity was estimated by the presence and intensity of bands on gels. The pooled samples were cleaned with Bioline SureClean Plus and/or via gel cuts before outsourcing library preparation to AITbiotech using TruSeq Nano DNA Library Preparation Kits (Illumina) or the Genome Institute of Singapore (GIS) using NEBNext DNA Library Preparation Kits (NEB). Paired-end sequencing was performed on Illumina Miseq (2×300-bp or 2×250-bp) or Hiseq 2500 platforms (2×250-bp) over multiple runs, thereby allowing troubleshooting and re-sequencing for specimens which initially failed to yield a sufficiently large numbers of reads. Some of the specimens were also sequenced on the MinION (Oxford Nanopore) platform using primers with a slightly longer tags (13-bp) and following the protocol described in Srivathsan et al. [57, 106]. Raw Illumina reads were processed with the bioinformatics pipeline and quality-control filters described in Meier et al. [55]. A BLAST search to GenBank’s nucleotide (nt) database was also conducted to identify and discard contaminants by parsing the BLAST output through *readsidentifier* [107] and removing barcodes with incorrect matches at >97% identity.

To obtain putative species units, the *cox1* barcodes were clustered over a range of uncorrected p-distance thresholds (2–4%) typically used for species delimitation in the literature [108]. The clustering was performed with a Python script (see Appendix) that implements the objective clustering algorithm of Meier et al. 2006 [59] and allows for large scale processing. USEARCH [60] (cluster_fast) was used to confirm the results by setting -id at 0.96, 0.97, and 0.98. To gauge how many of our species/specimens matched barcodes in public databases, we used the “Sequence ID” search of the Global Biodiversity Information Facility (GBIF). We then determined the number of matches with identity scores <97. We then counted the number of matches to barcodes with species-level identifications.

### Diversity analyses

For analysis of species richness and turnover, we initially focused on a core dataset of Diptera and Hymenoptera (62,066 specimens, 4002 species at 3% p-distance) that consisted of several taxa representing a range of ecological guilds (see Additional file 1: Table S1). This dataset included the sites that were sampled from 2012 to 2015 and overlap for April–September period in order to control for seasonal and sampling effects. We subsequently analyzed the full dataset consisting of all barcodes obtained in the project, which included more taxa and trapping sites. Results for the full dataset are described here because they are broadly congruent with those for the core dataset. The latter are in the supplementary. Community matrices were then generated with a Python script from the clustering outputs (clusterlist_to_commatrix.py: see Appendix). To assess the species richness of the six major habitat types, samples were rarefied with the *iNEXT* [109] R package (R Development Core Team) using 1,000 bootstrap replicates in order to account for unequal sampling completeness. The rarefaction was performed by coverage [61] in the main analysis (Fig. 1) and by specimen count in the supplementary (Additional file 2: Figure S3). Site comparisons were carried out by comparing species diversity post-rarefaction to the lowest coverage/smallest number of specimens. The habitat type “mangrove” was treated both as a single habitat as well as separate sites (PU, SB, SMN, SMO, others) in separate analyses. We also obtained species richness extrapolations for each habitat using a frequency ratio estimator [62] and a combined non-parametric estimator (CNE) [63] based on combined Chao1 and Chao2 extrapolations. Both were performed in R, with the former using the *breakaway* package and the latter using R code published with the original manuscript.

In order to study species turnover, we first excluded 11 trapping sites that had <100 specimens in total so as to prevent poor sampling from inflating site distinctness. We then determined the distinctness of the communities across habitats using non-metric multidimensional scaling (NMDS) plots that were prepared with PRIMER v7 [110] using Bray-Curtis dissimilarity. Plots were generated for each habitat type and the different mangrove sites; Bray-Curtis was chosen because it is a preferred choice for datasets that include abundance information. The dataset was split into three groups: dominant orders (Diptera and Hymenoptera) and all others combined. Community structuring by habitat was verified by multivariate analysis of variance (MANOVA) tests performed in R with the *manova* function, with habitat as the explanatory variable and the response variable being combined NMDS1 and NMDS2 coordinates, as well as both coordinates separately. The NMDS1 and NMDS2 coordinates were derived with the *metaMDS* function from *vegan* in R [111]. We also employ a model-based framework for testing if habitat influenced abundance distribution by coding the former as the explanatory variable and the latter as the response variable in *mvabund* [67] in R. The *manyglm* function was used to construct the model, with negative binomial error distribution. Lastly, analysis of similarities (ANOSIM) and similarity percentages (SIMPER) were performed in PRIMER under default parameters in order to obtain ANOSIM *p* values and R-statistics for both the entire dataset and the pairwise comparisons between habitat types. The SIMPER values were calculated for within and between-habitat types. The ANOSIM *p* values can be used to assess significant differences while the R-statistic allows for determining the degree of similarity, with values closer to 1 indicating greater distinctness. We also used the *betapart* [112] R package to examine if the observed dissimilarity (Bray-Curtis) was due to species turnover or nestedness. The *beta.multi.abund* and *beta.pair.abund* functions were used to split the global and pairwise dissimilarity scores into turnover and nestedness components. Lastly, the robustness of the results was tested by removing singleton, doubleton, and rare species (<5 and <10 individuals) from the datasets. The pruned datasets were subjected to the same analyses as the full dataset. For the guild-specific datasets, traps with fewer than three species were excluded in the species turnover analyses because large distances driven by undersampling can obscure signal.

To examine species turnover across larger geographic scales, dolichopodid, phorid, and mycetophilid specimens from Singapore were compared with those from Hong Kong (Dolichopodidae: 2,601; Phoridae: 562, Mycetophilidae: 186), and Brunei (Dolichopodidae: 2,800; Phoridae: 272), and data for the dolichopodids of Southern Thai mangroves (942 specimens). Since Singapore was more extensively sampled, the Singaporean dataset was randomly subsampled (10 iterations in Microsoft Excel with the RAND() function) to the number of specimens available for the other two countries (Additional file 1: Table S12). The species diversity after rarefaction was then compared (with 95% confidence intervals for the rarefied data).

### Ecological guild and plant diversity analyses

For the guild-level analysis, we focused on the core dataset consisting of the species belonging to the two dominant orders Diptera and Hymenoptera. This dataset consists of 62,066 specimens from 9 rainforest, 4 swamp forest, 4 urban forest, and 32 mangrove sites (Additional file 2: Figure S1). In order to test for an overall correlation between plant and insect diversity, we obtained data for the plant diversity in the respective sites from checklists and survey plots (Additional file 1: Table S8). In order to further examine the correlation between plant and insect diversity across multiple ecological guilds, we assigned the identified Diptera and Hymenoptera families and genera non-exclusively to ecological guilds (phytophages, pollinators, fungivores, parasitoids, predators, hematophages, and detritivores) based on known adult and larval natural history traits for the group (Additional file 1: Table S2). Taxa with different adult and larval natural histories are placed in both guilds. Taxa lacking sufficient information or with highly variable life-history strategies were assigned to the “Others/Unknown” category and excluded from analysis.

Barcodes from each guild were separately aligned and clustered at 3% p-distance. For taxa that have adults and immatures with different natural histories (i.e., belong to two distinct ecological guilds), the species counts were halved and placed into both guilds when calculating rarefied species abundance and richness. Guild distribution for each habitat was visualized with Voronoi treemaps via Proteomaps [113]. Species turnover for the guild-specific subsets were analyzed with PRIMER to generate NMDS plots, as well as ANOSIM and SIMPER values. The rarefied species richness values were also used for a multivariate model analysis. An ANCOVA model was constructed in R [114] with the *lm* function: insectdiv ~ site * habitat * guild * plantdiv, with insectdiv representing rarefied insect alpha-diversity and plantdiv representing plant species counts. The “site” factor was excluded due to collinearity, and the model was refined via stepwise removal of factors starting with the most complex (interaction terms) and least significant ones. At each stage, the *anova* function was used to assess loss of informational content and the final model was derived when the reported *p* value was significant (*p* < 0.05). The model’s residuals were examined to ensure the data were normal. Subsequently, the *anova* function from the *car* package [115] was used to obtain type-II test statistics. Finally, single-variable linear regression was performed in R with the *lm* function: *insectdiv ~ plantdiv* for each guild separately to obtain significance, multiple R-squared, and Spearman’s rho values.

### Supplementary Information

**Additional file 1: Table S1.** Specimen counts from full and core datasets. **Table S2.** Diptera and Hymenoptera taxa and their guild assignments. **Table S3.** Number and distribution of mOTUs. **Table S4.** Common and rare species found in 1 – 6 habitats. **Table S5.** Species turnover ANOSIM analysis with rare species removed. **Table S6.** Species turnover SIMPER analysis with rare species removed. **Table S7.** Species turnover and nestedness analysis with rare species removed. **Table S8.** Number of species of vascular plants in Singapore’s habitats. **Table S9.** Number of species from each guild, site and habitat type. **Table S10.** Species turnover ANOSIM analysis for each ecological guild. **Table S11.** Species turnover SIMPER analysis for each ecological guild. **Table S12.** Number of specimens from Singapore, Hong Kong and Brunei. **Table S13.** Collection periods and trap localities.

**Additional file 2: Figure S1.** Sampling locations in the Oriental realm and in Singapore. **Figure S2.** Arthropod orders sampled in this study and their species proportions. **Figure S3.** Insect alpha-diversity across tropical forest habitats. **Figure S4.** Breakaway and CNE species estimates for each habitat. **Figure S5.** Insect alpha-diversity across tropical forest habitats for the core dataset. **Figure S6.** Insect species turnover from the core dataset. **Figure S7.** Species diversity across habitats split by ecological guild. **Figure S8.** Species diversity across habitats (mangroves split) split by ecological guild. **Figure S9.** Species diversity and turnover from Singapore, Brunei, and Hong Kong datasets.

## Appendix

### Availability of data and materials

**Table:**

Reagent or resource	Source	Identifier
*Chemicals and reagents*		
QuickExtract	Lucigen	QE09050 and QE0905T
Tris-HCL	Promega	H5123
EDTA	J.T.Baker	8993-01
Sodium Hydroxide	Merck	S5158-1-100.01.04.07
PCR Master Mix	CWBio	CW0682L
Bovine Serum Albumin	HyClone	SH40015.01
SureClean Plus	Bioline	BIO-37048
Tagged primers	IDTDNA	See Srivathsan et al. [57]
Agarose A	Biobasic	DD0012
Gel-Red	Biotium	41003-1
*Software and algorithms*		
PEAR	Zhang et al. [116]	https://cme.h-its.org/exelixis/web/software/pear
miniBarcoder	Srivathsan et al. [57, 106]	https://github.com/asrivathsan/miniBarcoder; github commit 1536d02
BLAST 2.10.0	NCBI	https://blast.ncbi.nlm.nih.gov/Blast.cgi
Readsidentifier	Srivathsan et al. [107]	https://github.com/asrivathsan/readsidentifier; github commit 3bf39b9
Objective clustering	Meier et al. [59]	https://github.com/asrivathsan/obj_cluster; github commit 1d08240
USEARCH 11.0.667	Edgar [60]	https://www.drive5.com/usearch
Primer v7	Clarke & Gorley [110]	https://www.primer-e.com
Proteomaps 2.0	Liebermeister et al. [113]	https://bionic-vis.biologie.uni-greifswald.de
clusterlist_to_commatrix.py	This paper [117]	10.6084/m9.figshare.14706090.v1
Rstudio 1.0.153	R Development Core Team [114]	https://www.rstudio.com
R 3.4.1	R Development Core Team [114]	https://cran.r-project.org/bin/windows/base
R 4.0.3	R Development Core Team [114]	https://cran.r-project.org/bin/windows/base
R function CNE	Ronquist et al. [63]	https://github.com/ronquistlab/swedish-insect-fauna
R package iNEXT 2.0.20	Chao et al. [109]	https://cran.r-project.org/web/packages/iNEXT/index.html
R package ggplot2 3.3.3	Hadley [118]	https://cran.r-project.org/web/packages/ggplot2/index.html
R package vegan 2.5-7	Oksanen et al. [111]	https://cran.r-project.org/web/packages/vegan/index.html
R package car 3.0-10	Fox & Weisberg [115]	https://cran.r-project.org/web/packages/car/index.html
R package breakaway 3.0	Willis & Bunge [62]	https://cran.r-project.org/web/packages/breakaway/index.html
R package betapart 1.5.2	Baselga & Orme [112]	https://cran.r-project.org/web/packages/betapart/index.html
R package mvabund 4.1.9	Wang et al. [67]	https://cran.r-project.org/web/packages/mvabund/index.html
*Others*		
DNA barcode sequences	This paper [117]	10.6084/m9.figshare.14706090.v1
R commands	This paper [117]	10.6084/m9.figshare.14706090.v1
2003Community matrices	This paper [117]	10.6084/m9.figshare.14706090.v1
