# Supplementary material for: Mangroves are an overlooked hotspot of insect diversity despite low plant diversity
Source: BMC Biol. 2021 Sep 14;19:202. doi: 10.1186/s12915-021-01088-z (PMC8442405; doi:10.1186/s12915-021-01088-z)
Supplement: Supplementary file 1 — Additional file 1: Table S1. Specimen counts from full and core datasets. Table S2. Diptera and Hymenoptera taxa and their guild assignments. Table S3. Number and distribution of mOTUs. Table S4. Common and rare species found in 1 – 6 habitats. Table S5. Species turnover ANOSIM analysis with rare species removed. Table S6. Species turnover SIMPER analysis with rare species removed. Table S7. Species turnover and nestedness analysis with rare species removed. Table S8. Number of species of vascular plants in Singapore’s habitats. Table S9. Number of species from each guild, site and habitat type. Table S10. Species turnover ANOSIM analysis for each ecological guild. Table S11. Species turnover SIMPER analysis for each ecological guild. Table S12. Number of specimens from Singapore, Hong Kong and Brunei. Table S13. Collection periods and trap localities. [file 12915_2021_1088_MOESM1_ESM.docx]

**Table S1.** Specimen counts from the full dataset (core dataset shaded in green) with taxa split by habitat type and site (for mangroves only).

| Order | Family | Coastal Forest | Freshwater Swamp | Rainforest | Swamp Forest | Urban Forest | Mangrove PU | Mangrove SB | Mangrove SMN | Mangrove SMO | Mangrove Others |
| --- | --- | --- | --- | --- | --- | --- | --- | --- | --- | --- | --- |
| Araneae |  | 45 | 2 | 409 | 188 | 981 | 1080 | 782 | 320 | 544 | 172 |
| Blattodea | **Termitoidae** | 38 | 0 | 977 | 80 | 83 | 649 | 74 | 0 | 12 | 4 |
|  | **Other Blattodea** | 10 | 0 | 183 | 152 | 71 | 88 | 57 | 14 | 54 | 7 |
| Coleoptera |  | 54 | 26 | 915 | 574 | 0 | 92 | 78 | 57 | 265 | 2 |
| Dermaptera |  | 0 | 0 | 1 | 0 | 0 | 0 | 0 | 0 | 0 | 0 |
| Hemiptera |  | 76 | 10 | 2617 | 365 | 816 | 979 | 501 | 606 | 768 | 246 |
| Lepidoptera |  | 513 | 53 | 619 | 0 | 1 | 159 | 13 | 16 | 103 | 17 |
| Mantodea |  | 27 | 0 | 99 | 2 | 35 | 13 | 10 | 2 | 7 | 0 |
| Neuroptera | **Coniopterygidae** | 0 | 0 | 23 | 0 | 0 | 2 | 0 | 0 | 0 | 0 |
|  | **Other Neuroptera** | 0 | 0 | 1 | 0 | 0 | 0 | 0 | 0 | 0 | 0 |
| Orthoptera |  | 9 | 3 | 56 | 27 | 33 | 180 | 214 | 109 | 208 | 0 |
| Psocoptera |  | 141 | 15 | 283 | 0 | 0 | 97 | 2 | 0 | 0 | 1 |
| Trichoptera |  | 0 | 0 | 1 | 0 | 0 | 0 | 0 | 0 | 0 | 0 |
| Hymenoptera | **Formicidae** | 258 | 372 | 189 | 1055 | 3329 | 1406 | 292 | 789 | 730 | 85 |
|  | **Apoidea** | 302 | 289 | 2130 | 58 | 353 | 151 | 416 | 146 | 211 | 56 |
| Diptera | **Asilidae** | 14 | 2 | 29 | 12 | 9 | 105 | 33 | 45 | 73 | 41 |
|  | **Culicidae** | 226 | 3913 | 89 | 338 | 2 | 738 | 613 | 336 | 597 | 95 |
|  | **Dolichopodidae** | 1078 | 898 | 743 | 262 | 4143 | 7966 | 1945 | 7841 | 4070 | 1210 |
|  | **Empididae** | 77 | 7 | 41 | 106 | 69 | 472 | 18 | 1 | 0 | 3 |
|  | **Hybotidae** | 1 | 7 | 0 | 6 | 0 | 10 | 3 | 6 | 1 | 1 |
|  | **Keroplatidae** | 56 | 4 | 5 | 137 | 48 | 18 | 10 | 12 | 90 | 0 |
|  | **Mycetophilidae** | 202 | 272 | 874 | 1176 | 650 | 82 | 121 | 10 | 52 | 23 |
|  | **Phoridae** | 2406 | 2178 | 2122 | 345 | 6955 | 2242 | 808 | 293 | 302 | 308 |
|  | **Stratiomyidae** | 53 | 29 | 66 | 80 | 212 | 333 | 241 | 148 | 101 | 80 |
|  | **Syrphidae** | 34 | 28 | 19 | 10 | 6 | 150 | 141 | 14 | 36 | 43 |
|  | **Tabanidae** | 51 | 164 | 27 | 43 | 0 | 467 | 113 | 59 | 66 | 66 |
|  | **Tephritidae** | 4 | 0 | 42 | 21 | 8 | 397 | 70 | 652 | 607 | 13 |
|  | **Other Brachycera** | 1249 | 5840 | 1192 | 3574 | 2353 | 5444 | 1812 | 3148 | 3359 | 3485 |
|  | **Other Nematocera** | 2179 | 7894 | 1917 | 865 | 145 | 2154 | 46 | 34 | 65 | 403 |

**Table S2.** Diptera and Hymenoptera species used in the guild-level analyses are identified to higher taxonomic levels where possible and assigned to ecological guild based on known natural history traits (grey = guild assignment; some taxa are listed for several guilds because immatures and adults have different known natural histories).

| **Taxon** | | **Ecological Guild** | | | | | | | | | | | |
| --- | --- | --- | --- | --- | --- | --- | --- | --- | --- | --- | --- | --- | --- |
| **Family** | **Genus** | **Phytophages** | **Pollinators** | **Fungivores** | | **Parasitoids** | | **Predators** | **Haematophages** | | **Detritivores** | **Others/Unknown** | |
| **Diptera** | | | | | | | | | | | | | |
| **Agromyzidae** |  | ⌧ |  | |  | |  |  |  |  | | |  |
| **Anthomyiidae** |  |  |  | |  | |  |  |  |  | | | ⌧ |
| **Asilidae** |  |  |  | |  | |  | ⌧ |  |  | | |  |
| **Asteiidae** |  |  |  | | ⌧ | |  |  |  |  | | | ⌧ |
| **Athericidae** |  |  |  | |  | |  | ⌧ |  |  | | | ⌧ |
| **Bombyliidae** |  |  | ⌧ | |  | |  | ⌧ |  |  | | |  |
| **Calliphoridae** |  |  |  | |  | |  |  |  |  | | | ⌧ |
| **Canacidae** |  | ⌧ |  | |  | |  |  |  |  | | |  |
| **Chloropidae** | *Anacamptoneurum* | ⌧ |  | |  | |  |  |  |  | | |  |
| **Chloropidae** | *Cadrema* |  |  | |  | |  |  |  | ⌧ | | |  |
| **Chloropidae** | *Chlorops* | ⌧ |  | |  | |  |  |  |  | | |  |
| **Chloropidae** | *Chloropsina* | ⌧ |  | |  | |  |  |  |  | | |  |
| **Chloropidae** | *Conioscinella* | ⌧ |  | |  | |  |  |  |  | | |  |
| **Chloropidae** | *Dasyopa* |  |  | |  | |  |  |  |  | | | ⌧ |
| **Chloropidae** | *Gampsocera* |  |  | |  | |  |  |  |  | | | ⌧ |
| **Chloropidae** | *Gaurax* |  |  | |  | |  |  |  |  | | | ⌧ |
| **Chloropidae** | *Lasiambia* |  |  | |  | |  | ⌧ |  |  | | |  |
| **Chloropidae** | *Liohippelates* | ⌧ |  | |  | |  |  |  |  | | |  |
| **Chloropidae** | *Malloewia* |  |  | |  | |  |  |  |  | | | ⌧ |
| **Chloropidae** | *Olcella* | ⌧ |  | |  | |  |  |  |  | | |  |
| **Chloropidae** | *Oscinella* | ⌧ |  | |  | |  |  |  |  | | |  |
| **Chloropidae** | *Polyodaspis* |  |  | |  | |  | ⌧ |  |  | | |  |
| **Chloropidae** | *Pseudogaurax* |  |  | |  | |  | ⌧ |  |  | | |  |
| **Taxon** | | **Ecological Guild** | | | | | | | | | | | |
| **Family** | **Genus** | **Phytophages** | **Pollinators** | | **Fungivores** | | **Parasitoids** | **Predators** | **Haematophages** | **Detritivores** | | | **Others/Unknown** |
| **Chloropidae** | *Pseudopachychaeta* | ⌧ |  | |  | |  |  |  |  | | |  |
| **Chloropidae** | *Rhodesiella* |  |  | |  | |  |  |  | ⌧ | | |  |
| **Chloropidae** | *Thaumatomyia* |  |  | |  | |  | ⌧ |  |  | | |  |
| **Chloropidae** | *Thyridula* |  |  | |  | |  |  |  |  | | | ⌧ |
| **Chloropidae** | *Tricimba* |  |  | |  | |  |  |  | ⌧ | | |  |
| **Clusiidae** |  | ⌧ |  | |  | |  |  |  |  | | | ⌧ |
| **Coelopidae** |  | ⌧ |  | |  | |  |  |  |  | | |  |
| **Cryptochetidae** |  |  |  | |  | | ⌧ |  |  |  | | |  |
| **Culicidae** |  |  |  | |  | |  |  | ⌧ | ⌧ | | |  |
| **Diastatidae** |  |  |  | |  | |  |  |  | ⌧ | | |  |
| **Diopsidae** |  | ⌧ |  | |  | |  |  |  | ⌧ | | |  |
| **Dolichopodidae** |  |  |  | |  | |  | ⌧ |  |  | | |  |
| **Drosophilidae** | *Apenthecia* | ⌧ |  | |  | |  |  |  |  | | |  |
| **Drosophilidae** | *Chymomyza* | ⌧ |  | |  | |  |  |  |  | | |  |
| **Drosophilidae** | *Colocasiomyia* | ⌧ |  | |  | |  |  |  |  | | |  |
| **Drosophilidae** | *Dichaetophora* |  |  | | ⌧ | |  |  |  |  | | |  |
| **Drosophilidae** | *Drosophila* |  |  | |  | |  |  |  |  | | | ⌧ |
| **Drosophilidae** | *Gitona* | ⌧ |  | |  | |  |  |  |  | | |  |
| **Drosophilidae** | *Hirtodrosophila* |  |  | | ⌧ | |  |  |  |  | | |  |
| **Drosophilidae** | *Hypselothyrea* | ⌧ |  | |  | |  |  |  |  | | |  |
| **Drosophilidae** | *Leucophenga* | ⌧ |  | |  | |  |  |  |  | | |  |
| **Drosophilidae** | *Liodrosophila* | ⌧ |  | |  | |  |  |  |  | | |  |
| **Drosophilidae** | *Luzonimyia* |  |  | |  | |  |  |  |  | | | ⌧ |
| **Drosophilidae** | *Microdrosophila* | ⌧ |  | |  | |  |  |  |  | | |  |
| **Drosophilidae** | *Mycodrosophila* |  |  | | ⌧ | |  |  |  |  | | |  |
| **Drosophilidae** | *Paramycodrosophila* |  |  | | ⌧ | |  |  |  |  | | |  |
| **Drosophilidae** | *Scaptodrosophila* | ⌧ |  | |  | |  |  |  |  | | |  |
| **Drosophilidae** | *Scaptomyza* | ⌧ |  | |  | |  |  |  |  | | |  |
| **Taxon** | | **Ecological Guild** | | | | | | | | | | | |
| **Family** | **Genus** | **Phytophages** | **Pollinators** | | **Fungivores** | | **Parasitoids** | **Predators** | **Haematophages** | **Detritivores** | | | **Others/Unknown** |
| **Drosophilidae** | *Stegana* | ⌧ |  | |  | |  |  |  |  | | |  |
| **Drosophilidae** | *Zaprionus* | ⌧ |  | |  | |  |  |  |  | | |  |
| **Empididae** |  |  |  | |  | |  | ⌧ |  |  | | |  |
| **Ephydridae** | *Allotrichoma* |  |  | |  | |  |  |  | ⌧ | | |  |
| **Ephydridae** | *Atissa* |  |  | |  | |  |  |  | ⌧ | | |  |
| **Ephydridae** | *Brachydeutera* | ⌧ |  | |  | |  |  |  |  | | |  |
| **Ephydridae** | *Cerobothrium* |  |  | |  | |  |  |  |  | | | ⌧ |
| **Ephydridae** | *Ceropsilopa* | ⌧ |  | |  | |  |  |  |  | | |  |
| **Ephydridae** | *Discocerina* | ⌧ |  | |  | |  |  |  |  | | |  |
| **Ephydridae** | *Donaceus* |  |  | |  | |  |  |  |  | | | ⌧ |
| **Ephydridae** | *Glenanthe* |  |  | |  | |  |  |  |  | | | ⌧ |
| **Ephydridae** | *Hecamedoides* |  |  | |  | |  |  |  |  | | | ⌧ |
| **Ephydridae** | *Hydrellia* | ⌧ |  | |  | |  |  |  |  | | |  |
| **Ephydridae** | *Limnellia* |  |  | |  | |  |  |  |  | | | ⌧ |
| **Ephydridae** | *Nostima* | ⌧ |  | |  | |  |  |  |  | | |  |
| **Ephydridae** | *Notiphila* |  |  | |  | |  |  |  | ⌧ | | |  |
| **Ephydridae** | *Ochthera* |  |  | |  | |  | ⌧ |  |  | | |  |
| **Ephydridae** | *Orasiopa* |  |  | |  | |  |  |  |  | | | ⌧ |
| **Ephydridae** | *Paralimna* | ⌧ |  | |  | |  |  |  |  | | |  |
| **Ephydridae** | *Placopsidella* |  |  | |  | |  | ⌧ |  |  | | |  |
| **Ephydridae** | *Polytrichophora* |  |  | |  | |  |  |  |  | | | ⌧ |
| **Ephydridae** | *Ptilomyia* |  |  | |  | |  |  |  |  | | | ⌧ |
| **Ephydridae** | *Rhynchopsilopa* |  |  | |  | |  |  |  |  | | | ⌧ |
| **Ephydridae** | *Trimerogastra* |  |  | |  | |  |  |  |  | | | ⌧ |
| **Ephydridae** | *Trypetomima* |  |  | |  | |  |  |  |  | | | ⌧ |
| **Ephydridae** | *Zeros* |  |  | |  | |  |  |  |  | | | ⌧ |
| **Hybotidae** |  |  |  | |  | |  | ⌧ |  |  | | |  |
| **Keroplatidae** |  | ⌧ |  | | ⌧ | |  |  |  |  | | |  |
| **Taxon** | | **Ecological Guild** | | | | | | | | | | | |
| **Family** | **Genus** | **Phytophages** | **Pollinators** | | **Fungivores** | | **Parasitoids** | **Predators** | **Haematophages** | **Detritivores** | | | **Others/Unknown** |
| **Lauxaniidae** |  | ⌧ |  | |  | |  |  |  |  | | |  |
| **Lonchaeidae** |  | ⌧ |  | |  | |  |  |  |  | | |  |
| **Lygistorrhinidae** |  | ⌧ |  | | ⌧ | |  |  |  |  | | |  |
| **Megamerinidae** |  |  |  | |  | |  | ⌧ |  |  | | |  |
| **Micropezidae** |  |  |  | |  | |  |  |  |  | | | ⌧ |
| **Milichiidae** | *Aldrichiomyza* |  |  | |  | |  |  |  |  | | | ⌧ |
| **Milichiidae** | *Leptometopa* |  |  | |  | |  |  |  | ⌧ | | |  |
| **Milichiidae** | *Milichia* |  |  | |  | |  |  |  |  | | | ⌧ |
| **Milichiidae** | *Milichiella* |  |  | |  | |  |  |  | ⌧ | | |  |
| **Milichiidae** | *Neophyllomyza* | ⌧ |  | |  | |  |  |  |  | | |  |
| **Milichiidae** | *Paramyia* |  |  | |  | |  |  |  |  | | | ⌧ |
| **Milichiidae** | *Phyllomyza* |  |  | |  | |  |  |  | ⌧ | | |  |
| **Muscidae** |  |  |  | |  | |  |  |  |  | | | ⌧ |
| **Mycetophilidae** |  | ⌧ |  | | ⌧ | |  |  |  |  | | |  |
| **Neriidae** |  | ⌧ |  | |  | |  |  |  |  | | | ⌧ |
| **Odiniidae** |  |  |  | |  | |  |  |  |  | | | ⌧ |
| **Periscelididae** |  | ⌧ |  | |  | |  |  |  |  | | |  |
| **Phoridae** |  |  |  | |  | |  |  |  |  | | | ⌧ |
| **Pipunculidae** |  |  |  | |  | | ⌧ |  |  |  | | |  |
| **Platypezidae** |  |  |  | | ⌧ | |  |  |  | ⌧ | | |  |
| **Platystomatidae** |  |  |  | |  | |  |  |  |  | | | ⌧ |
| **Psilidae** |  | ⌧ |  | |  | |  |  |  |  | | |  |
| **Pyrgotidae** |  |  |  | |  | | ⌧ |  |  |  | | |  |
| **Rhagionidae** |  |  |  | |  | |  | ⌧ | ⌧ |  | | |  |
| **Rhiniidae** |  |  |  | |  | |  | ⌧ |  |  | | |  |
| **Sarcophagidae** |  |  |  | |  | |  |  |  |  | | | ⌧ |
| **Sciaridae** |  | ⌧ |  | | ⌧ | |  |  |  |  | | |  |
| **Sphaeroceridae** |  |  |  | |  | |  |  |  |  | | | ⌧ |
| **Taxon** | | **Ecological Guild** | | | | | | | | | | | |
| **Family** | **Genus** | **Phytophages** | **Pollinators** | | **Fungivores** | | **Parasitoids** | **Predators** | **Haematophages** | **Detritivores** | | | **Others/Unknown** |
| **Stratiomyiidae** |  | ⌧ |  | |  | |  |  |  | ⌧ | | |  |
| **Syrphidae** | *Allobaccha* |  | ⌧ | |  | |  | ⌧ |  |  | | |  |
| **Syrphidae** | *Allograpta* |  | ⌧ | |  | |  | ⌧ |  |  | | |  |
| **Syrphidae** | *Asarkina* |  | ⌧ | |  | |  | ⌧ |  |  | | |  |
| **Syrphidae** | *Ceriana* |  | ⌧ | |  | |  |  |  | ⌧ | | |  |
| **Syrphidae** | *Eosmallota* |  | ⌧ | |  | |  |  |  | ⌧ | | |  |
| **Syrphidae** | *Eristalinus* |  | ⌧ | |  | |  |  |  | ⌧ | | |  |
| **Syrphidae** | *Eristalis* |  | ⌧ | |  | |  |  |  | ⌧ | | |  |
| **Syrphidae** | *Eumerus* |  | ⌧ | |  | |  |  |  | ⌧ | | |  |
| **Syrphidae** | *Graptomyza* |  | ⌧ | |  | |  |  |  | ⌧ | | |  |
| **Syrphidae** | *Ischiodon* |  | ⌧ | |  | |  | ⌧ |  |  | | |  |
| **Syrphidae** | *Microdon* |  | ⌧ | |  | |  | ⌧ |  |  | | |  |
| **Syrphidae** | *Paragus* |  | ⌧ | |  | |  | ⌧ |  |  | | |  |
| **Syrphidae** | *Psilota* |  | ⌧ | |  | |  |  |  | ⌧ | | |  |
| **Syrphidae** | *Spheginobaccha* |  | ⌧ | |  | |  |  |  |  | | | ⌧ |
| **Syrphidae** | *Syritta* |  | ⌧ | |  | |  |  |  | ⌧ | | |  |
| **Syrphidae** | *Volucella* |  | ⌧ | |  | |  |  |  | ⌧ | | |  |
| **Tabanidae** |  |  |  | |  | |  | ⌧ | ⌧ |  | | |  |
| **Tachinidae** |  |  |  | |  | | ⌧ |  |  |  | | | ⌧ |
| **Tephritidae** |  | ⌧ |  | |  | |  |  |  |  | | |  |
| **Ulidiidae** |  |  |  | |  | |  |  |  |  | | | ⌧ |
| **Xenasteiidae** |  |  |  | |  | |  |  |  |  | | | ⌧ |
| **Xylomyidae** |  |  |  | |  | |  |  |  |  | | | ⌧ |
| **Hymenoptera** | | | | | | | | | | | | | |
| **Aphelinidae** |  |  |  | |  | | ⌧ |  |  |  | | |  |
| **Apidae** |  |  | ⌧ | |  | |  |  |  |  | | |  |
| **Bethylidae** |  |  |  | |  | | ⌧ | ⌧ |  |  | | |  |
| **Braconidae** |  |  |  | |  | | ⌧ |  |  |  | | |  |
| **Taxon** | | **Ecological Guild** | | | | | | | | | | | |
| **Family** | **Genus** | **Phytophages** | **Pollinators** | | **Fungivores** | | **Parasitoids** | **Predators** | **Haematophages** | **Detritivores** | | | **Others/Unknown** |
| **Ceraphronidae** |  |  |  | |  | | ⌧ |  |  |  | | |  |
| **Chalcidae** |  |  |  | |  | | ⌧ |  |  |  | | |  |
| **Chrysididae** |  |  |  | |  | | ⌧ |  |  |  | | |  |
| **Colletidae** |  |  | ⌧ | |  | |  |  |  |  | | |  |
| **Crabronidae** |  |  |  | |  | |  |  |  |  | | | ⌧ |
| **Diapriidae** |  |  |  | |  | | ⌧ |  |  |  | | |  |
| **Dryinidae** |  |  |  | |  | | ⌧ |  |  |  | | |  |
| **Eulophidae** |  |  |  | |  | | ⌧ |  |  |  | | |  |
| **Eupelmidae** |  |  |  | |  | | ⌧ |  |  |  | | |  |
| **Evaniidae** |  |  |  | |  | | ⌧ |  |  |  | | |  |
| **Figitidae** |  |  |  | |  | | ⌧ |  |  |  | | |  |
| **Formicidae** | *Acropyga* |  |  | |  | |  |  |  |  | | | ⌧ |
| **Formicidae** | *Anochetus* |  |  | |  | |  | ⌧ |  |  | | |  |
| **Formicidae** | *Anoplolepis* |  |  | |  | |  | ⌧ |  |  | | |  |
| **Formicidae** | *Aphaenogaster* |  |  | |  | |  |  |  |  | | | ⌧ |
| **Formicidae** | *Brachyponera* |  |  | |  | |  | ⌧ |  |  | | |  |
| **Formicidae** | *Camponotus* |  |  | |  | |  |  |  |  | | | ⌧ |
| **Formicidae** | *Cardiocondyla* |  |  | |  | |  |  |  |  | | | ⌧ |
| **Formicidae** | *Carebara* |  |  | |  | |  | ⌧ |  |  | | |  |
| **Formicidae** | *Cataulacus* |  |  | |  | |  |  |  |  | | | ⌧ |
| **Formicidae** | *Chronoxenus* |  |  | |  | |  |  |  |  | | | ⌧ |
| **Formicidae** | *Colobopsis* |  |  | |  | |  |  |  |  | | | ⌧ |
| **Formicidae** | *Crematogaster* |  |  | |  | |  |  |  |  | | | ⌧ |
| **Formicidae** | *Cryptopone* |  |  | |  | |  |  |  |  | | | ⌧ |
| **Formicidae** | *Diacamma* |  |  | |  | |  | ⌧ |  |  | | |  |
| **Formicidae** | *Discothyrea* |  |  | |  | |  | ⌧ |  |  | | |  |
| **Formicidae** | *Dolichoderus* |  |  | |  | |  |  |  |  | | | ⌧ |
| **Formicidae** | *Echinopla* |  |  | |  | |  |  |  |  | | | ⌧ |
| **Taxon** | | **Ecological Guild** | | | | | | | | | | | |
| **Family** | **Genus** | **Phytophages** | **Pollinators** | | **Fungivores** | | **Parasitoids** | **Predators** | **Haematophages** | **Detritivores** | | | **Others/Unknown** |
| **Formicidae** | *Ectomomyrmex* |  |  | |  | |  | ⌧ |  |  | | |  |
| **Formicidae** | *Euponera* |  |  | |  | |  | ⌧ |  |  | | |  |
| **Formicidae** | *Euprenolepis* |  |  | |  | |  |  |  |  | | | ⌧ |
| **Formicidae** | *Gauromyrmex* |  |  | |  | |  |  |  |  | | | ⌧ |
| **Formicidae** | *Hypoponera* |  |  | |  | |  | ⌧ |  |  | | |  |
| **Formicidae** | *Iridomyrmex* |  |  | |  | |  |  |  |  | | | ⌧ |
| **Formicidae** | *Leptogenys* |  |  | |  | |  |  |  |  | | | ⌧ |
| **Formicidae** | *Lioponera* |  |  | |  | |  | ⌧ |  |  | | |  |
| **Formicidae** | *Mayriella* |  |  | |  | |  |  |  |  | | | ⌧ |
| **Formicidae** | *Meranoplus* |  |  | |  | |  |  |  |  | | | ⌧ |
| **Formicidae** | *Mesoponera* |  |  | |  | |  |  |  |  | | | ⌧ |
| **Formicidae** | *Monomorium* |  |  | |  | |  |  |  |  | | | ⌧ |
| **Formicidae** | *Myrmecina* |  |  | |  | |  | ⌧ |  |  | | |  |
| **Formicidae** | *Nylanderia* |  |  | |  | |  |  |  |  | | | ⌧ |
| **Formicidae** | *Odontomachus* |  |  | |  | |  |  |  |  | | | ⌧ |
| **Formicidae** | *Odontoponera* |  |  | |  | |  |  |  |  | | | ⌧ |
| **Formicidae** | *Oecophylla* |  |  | |  | |  |  |  |  | | | ⌧ |
| **Formicidae** | *Paraparatrechina* |  |  | |  | |  |  |  |  | | | ⌧ |
| **Formicidae** | *Paratopula* |  |  | |  | |  |  |  |  | | | ⌧ |
| **Formicidae** | *Paratrechina* |  |  | |  | |  |  |  |  | | | ⌧ |
| **Formicidae** | *Pheidole* |  |  | |  | |  |  |  |  | | | ⌧ |
| **Formicidae** | *Philidris* | ⌧ |  | |  | |  |  |  |  | | |  |
| **Formicidae** | *Platythyrea* |  |  | |  | |  | ⌧ |  |  | | |  |
| **Formicidae** | *Polyrhachis* | ⌧ |  | |  | |  |  |  |  | | |  |
| **Formicidae** | *Ponera* |  |  | |  | |  |  |  |  | | | ⌧ |
| **Formicidae** | *Prenolepis* |  |  | |  | |  |  |  |  | | | ⌧ |
| **Formicidae** | *Prionopelta* |  |  | |  | |  |  |  |  | | | ⌧ |
| **Formicidae** | *Proatta* |  |  | |  | |  | ⌧ |  |  | | |  |
| **Taxon** | | **Ecological Guild** | | | | | | | | | | | |
| **Family** | **Genus** | **Phytophages** | **Pollinators** | | **Fungivores** | | **Parasitoids** | **Predators** | **Haematophages** | **Detritivores** | | | **Others/Unknown** |
| **Formicidae** | *Probolomyrmex* |  |  | |  | |  |  |  |  | | | ⌧ |
| **Formicidae** | *Pseudoneoponera* |  |  | |  | |  | ⌧ |  |  | | |  |
| **Formicidae** | *Strumigenys* |  |  | |  | |  | ⌧ |  |  | | |  |
| **Formicidae** | *Rhopalomastix* |  |  | |  | |  | ⌧ |  |  | | |  |
| **Formicidae** | *Solenopsis* |  |  | |  | |  |  |  |  | | | ⌧ |
| **Formicidae** | *Stigmatomma* |  |  | |  | |  | ⌧ |  |  | | |  |
| **Formicidae** | *Strumigenys* |  |  | |  | |  | ⌧ |  |  | | |  |
| **Formicidae** | *Tapinoma* |  |  | |  | |  |  |  |  | | | ⌧ |
| **Formicidae** | *Technomyrmex* |  |  | |  | |  |  |  |  | | | ⌧ |
| **Formicidae** | *Tetramorium* |  |  | |  | |  | ⌧ |  |  | | |  |
| **Formicidae** | *Tetraponera* | ⌧ |  | |  | |  |  |  |  | | |  |
| **Formicidae** | *Vollenhovia* |  |  | |  | |  |  |  |  | | | ⌧ |
| **Halictidae** |  |  | ⌧ | |  | |  |  |  |  | | |  |
| **Ichneumonidae** |  |  |  | |  | | ⌧ |  |  |  | | |  |
| **Megachilidae** |  |  | ⌧ | |  | |  |  |  |  | | |  |
| **Mymaridae** |  |  |  | |  | | ⌧ |  |  |  | | |  |
| **Platygastridae** |  |  |  | |  | | ⌧ |  |  |  | | |  |
| **Pompilidae** |  |  |  | |  | | ⌧ |  |  |  | | |  |
| **Pteromalidae** |  |  |  | |  | | ⌧ |  |  |  | | |  |
| **Scoliidae** |  |  |  | |  | | ⌧ |  |  |  | | |  |
| **Sphecidae** |  |  |  | |  | | ⌧ |  |  |  | | |  |
| **Sphecidae** |  |  |  | |  | |  | ⌧ |  |  | | |  |
| **Tiphiidae** |  |  |  | |  | | ⌧ |  |  |  | | |  |
| **Trichogrammatidae** |  |  |  | |  | | ⌧ |  |  |  | | |  |
| **Vespidae** |  |  | ⌧ | |  | |  | ⌧ |  |  | | |  |

**Table S3.** Number and distribution of mOTUs delimited using different thresholds (144,865 barcoded specimens)

| Habitat/Country | No. of Barcodes | No. of mOTUs from Objective Clustering | | | No. of mOTUs from USEARCH | | |
| --- | --- | --- | --- | --- | --- | --- | --- |
|  |  | **2%** | **3%** | **4%** | **id=0.98** | **id=0.97** | **id=0.96** |
| Singapore full dataset | | | | | | | |
| Mangroves | 67058 | 3557 | 3437 | 3320 | 3710 | 3524 | 3436 |
| Rainforest | 15681 | 2625 | 2573 | 2539 | 2669 | 2603 | 2570 |
| Swamp forest | 9469 | 1843 | 1804 | 1753 | 1895 | 1828 | 1795 |
| Urban forest | 20243 | 1552 | 1515 | 1478 | 1616 | 1549 | 1510 |
| Freshwater swamp | 22046 | 1881 | 1812 | 1744 | 1988 | 1878 | 1805 |
| Coastal forest | 9859 | 1707 | 1667 | 1627 | 1755 | 1691 | 1664 |
| Total | **144356** | **8903** | **8572** | **8256** | **9315** | **8821** | **8520** |
| Subset used for guild-level analysis | | | | | | | |
| Mangroves | 37641 | 1778 | 1720 | 1673 | 1828 | 1744 | 1702 |
| Rainforest | 9212 | 1525 | 1490 | 1474 | 1545 | 1503 | 1483 |
| Swamp forest | 5893 | 1090 | 1052 | 1030 | 1105 | 1070 | 1048 |
| Urban forest | 9320 | 919 | 898 | 885 | 941 | 908 | 893 |
| Total | **62066** | **4169** | **4002** | **3917** | **4298** | **4098** | **3994** |
| Southeast and East Asian datasets | | | | | | | |
| *Dolichopodidae* | | | | | | | |
| Singapore | 17860 | 263 | 254 | 248 | 280 | 259 | 249 |
| Hong Kong | 2601 | 111 | 109 | 104 | 115 | 110 | 106 |
| Brunei | 2800 | 98 | 96 | 95 | 107 | 98 | 95 |
| Thailand | 924 | 80 | 74 | 72 | 93 | 80 | 73 |
| Total | **24185** | **480** | **453** | **426** | **543** | **482** | **447** |
| *Phoridae* | | | | | | | |
| Singapore | 2134 | 293 | 281 | 278 | 300 | 285 | 280 |
| Hong Kong | 562 | 137 | 129 | 125 | 138 | 130 | 129 |
| Brunei | 272 | 76 | 76 | 75 | 77 | 76 | 75 |
| Total | **2968** | **453** | **429** | **417** | **467** | **437** | **431** |
| *Mycetophilidae* | | | | | | | |
| Singapore | 223 | 45 | 44 | 43 | 45 | 44 | 44 |
| Hong Kong | 186 | 26 | 25 | 25 | 26 | 25 | 25 |
| Total | **409** | **69** | **67** | **67** | **70** | **67** | **67** |

**Table S4.** Common and rare species found in only 1, 2, 3, 4, 5 or all habitats.

|  | No. of species | | | | |
| --- | --- | --- | --- | --- | --- |
|  | **Full dataset** | **No singletons** | **No doubletons** | **No species with <5 specimens** | **No species with <10 specimens** |
| Species in mangroves only | 1788 | 880 | 638 | 441 | 256 |
| Species in rainforests only | 1569 | 638 | 415 | 243 | 91 |
| Species in swamp forests only | 875 | 342 | 200 | 102 | 39 |
| Species in urban forests only | 509 | 166 | 101 | 58 | 25 |
| Species in freshwater swamps only | 794 | 360 | 237 | 127 | 56 |
| Species in coastal forests only | 454 | 153 | 71 | 33 | 14 |
| Species in two habitats | 1580 | 1580 | 1253 | 887 | 555 |
| Species in three habitats | 565 | 565 | 565 | 494 | 350 |
| Species in four habitats | 274 | 274 | 274 | 265 | 230 |
| Species in five habitats | 116 | 116 | 116 | 116 | 109 |
| Species in all habitats | 48 | 48 | 48 | 48 | 48 |
| Total | **8572** | **5122** | **3918** | **2814** | **1773** |

**Table S5.** Species turnover ANOSIM analysis results indicate distinct communities in each habitat type, whether with singletons and doubletons removed, or species with less than 5 and 10 specimens. Pairwise p-value outputs are displayed in the bottom-left of the pairwise matrix while the R-statistics are displayed at the top-right.

**No Singletons**

| **Overall P:** 0.001 **Overall R:** 0.777 | | | | |  |  |
| --- | --- | --- | --- | --- | --- | --- |
|  | **Rainforest** | **Urban forest** | **Swamp forest** | **Mangrove** | **Freshwater swamp** | **Coastal forest** |
| **Rainforest** |  | 0.809 | 0.981 | 0.948 | 0.972 | 0.951 |
| **Urban forest** | 0.001 |  | 0.747 | 0.815 | 0.571 | 0.173 |
| **Swamp forest** | 0.001 | 0.001 |  | 0.927 | 0.756 | 0.893 |
| **Mangrove** | 0.001 | 0.001 | 0.001 |  | 0.852 | 0.541 |
| **Freshwater swamp** | 0.001 | 0.001 | 0.008 | 0.001 |  | 0.347 |
| **Coastal forest** | 0.001 | 0.083 | 0.005 | 0.001 | 0.017 |  |

**No Doubletons**

| **Overall P:** 0.001 **Overall R:** 0.774 | | | | |  |  |
| --- | --- | --- | --- | --- | --- | --- |
|  | **Rainforest** | **Urban forest** | **Swamp forest** | **Mangrove** | **Freshwater swamp** | **Coastal forest** |
| **Rainforest** |  | 0.803 | 0.980 | 0.946 | 0.972 | 0.954 |
| **Urban forest** | 0.001 |  | 0.735 | 0.816 | 0.563 | 0.179 |
| **Swamp forest** | 0.001 | 0.001 |  | 0.922 | 0.750 | 0.889 |
| **Mangrove** | 0.001 | 0.001 | 0.001 |  | 0.849 | 0.538 |
| **Freshwater swamp** | 0.001 | 0.001 | 0.008 | 0.001 |  | 0.331 |
| **Coastal forest** | 0.002 | 0.072 | 0.005 | 0.001 | 0.019 |  |

**No Species <5 Specimens**

| **Overall P:** 0.001 **Overall R:** 0.767 | | | | |  |  |
| --- | --- | --- | --- | --- | --- | --- |
|  | **Rainforest** | **Urban forest** | **Swamp forest** | **Mangrove** | **Freshwater swamp** | **Coastal forest** |
| **Rainforest** |  | 0.795 | 0.970 | 0.941 | 0.971 | 0.954 |
| **Urban forest** | 0.001 |  | 0.720 | 0.817 | 0.559 | 0.180 |
| **Swamp forest** | 0.001 | 0.001 |  | 0.913 | 0.750 | 0.885 |
| **Mangrove** | 0.001 | 0.001 | 0.001 |  | 0.843 | 0.533 |
| **Freshwater swamp** | 0.002 | 0.001 | 0.008 | 0.001 |  | 0.331 |
| **Coastal forest** | 0.002 | 0.061 | 0.005 | 0.001 | 0.017 |  |

**No Species <10 Specimens**

| **Overall P:** 0.001 **Overall R:** 0.759 | | | | |  |  |
| --- | --- | --- | --- | --- | --- | --- |
|  | **Rainforest** | **Urban forest** | **Swamp forest** | **Mangrove** | **Freshwater swamp** | **Coastal forest** |
| **Rainforest** |  | 0.779 | 0.959 | 0.934 | 0.967 | 0.952 |
| **Urban forest** | 0.001 |  | 0.701 | 0.819 | 0.548 | 0.178 |
| **Swamp forest** | 0.001 | 0.002 |  | 0.904 | 0.738 | 0.877 |
| **Mangrove** | 0.001 | 0.001 | 0.001 |  | 0.837 | 0.526 |
| **Freshwater swamp** | 0.002 | 0.001 | 0.008 | 0.001 |  | 0.331 |
| **Coastal forest** | 0.001 | 0.062 | 0.005 | 0.001 | 0.017 |  |

**Table S6.** Species turnover SIMPER analysis results indicate distinct communities in each habitat type, whether with singletons and doubletons removed, or species with less than 5 and 10 specimens.

**No Singletons**

|  | **Within habitat (%)** | **Between habitats (%)** | | | | | |
| --- | --- | --- | --- | --- | --- | --- | --- |
|  |  | **Rain- forest** | **Urban forest** | **Swamp forest** | **Mangrove** | **Fresh-water swamp** | **Coastal forest** |
| **Rainforest** | 33.65 |  |  |  |  |  |  |
| **Urban forest** | 13.70 | 3.57 |  |  |  |  |  |
| **Swamp forest** | 35.74 | 15.86 | 3.31 |  |  |  |  |
| **Mangrove** | 12.78 | 1.80 | 3.26 | 2.22 |  |  |  |
| **Freshwater swamp** | 18.80 | 2.36 | 5.06 | 4.57 | 2.93 |  |  |
| **Coastal forest** | 12.98 | 4.27 | 10.04 | 4.50 | 6.44 | 9.82 |  |

**No Doubletons**

|  | **Within habitat (%)** | **Between habitats (%)** | | | | | |
| --- | --- | --- | --- | --- | --- | --- | --- |
|  |  | **Rain- forest** | **Urban forest** | **Swamp forest** | **Mangrove** | **Fresh-water swamp** | **Coastal forest** |
| **Rainforest** | 35.83 |  |  |  |  |  |  |
| **Urban forest** | 14.15 | 3.79 |  |  |  |  |  |
| **Swamp forest** | 38.05 | 17.12 | 3.55 |  |  |  |  |
| **Mangrove** | 13.14 | 1.91 | 3.36 | 2.39 |  |  |  |
| **Freshwater swamp** | 19.61 | 2.52 | 5.30 | 4.90 | 3.07 |  |  |
| **Coastal forest** | 13.62 | 4.57 | 10.44 | 4.86 | 6.68 | 10.37 |  |

**No Species <5 Specimens**

|  | **Within habitat (%)** | **Between habitats (%)** | | | | | |
| --- | --- | --- | --- | --- | --- | --- | --- |
|  |  | **Rain- forest** | **Urban forest** | **Swamp forest** | **Mangrove** | **Fresh-water swamp** | **Coastal forest** |
| **Rainforest** | 38.68 |  |  |  |  |  |  |
| **Urban forest** | 14.86 | 4.13 |  |  |  |  |  |
| **Swamp forest** | 40.06 | 18.89 | 3.93 |  |  |  |  |
| **Mangrove** | 13.65 | 2.08 | 3.50 | 2.65 |  |  |  |
| **Freshwater swamp** | 20.84 | 2.76 | 5.68 | 5.46 | 3.29 |  |  |
| **Coastal forest** | 14.49 | 4.99 | 11.08 | 5.39 | 7.03 | 11.15 |  |

**No Species <10 Specimens**

|  | **Within habitat (%)** | **Between habitats (%)** | | | | | |
| --- | --- | --- | --- | --- | --- | --- | --- |
|  |  | **Rain- forest** | **Urban forest** | **Swamp forest** | **Mangrove** | **Fresh-water swamp** | **Coastal forest** |
| **Rainforest** | 42.79 |  |  |  |  |  |  |
| **Urban forest** | 15.91 | 4.79 |  |  |  |  |  |
| **Swamp forest** | 42.93 | 21.56 | 4.49 |  |  |  |  |
| **Mangrove** | 14.55 | 2.41 | 3.75 | 3.04 |  |  |  |
| **Freshwater swamp** | 22.41 | 3.25 | 6.28 | 6.25 | 3.63 |  |  |
| **Coastal forest** | 15.95 | 5.83 | 12.09 | 6.19 | 7.65 | 12.16 |  |

**Table S7.** Species turnover and nestedness analysis reveal that the high dissimilarity is due more to turnover rather than nestedness, whether with singletons and doubletons removed, or species with less than 5 and 10 specimens. Pairwise turnover values are displayed in the bottom-left of the pairwise matrix while the nestedness values are in the top-right.

**No Singletons**

| **Overall Dissimilarity:** 0.944 **Overall** **Turnover:** 0.894 **Overall Nestedness:** 0.051 | | | | | | |
| --- | --- | --- | --- | --- | --- | --- |
|  | **Rainforest** | **Urban forest** | **Swamp forest** | **Mangrove** | **Freshwater swamp** | **Coastal forest** |
| **Rainforest** |  | 0.013 | 0.075 | 0.058 | 0.009 | 0.020 |
| **Urban forest** | 0.911 |  | 0.031 | 0.099 | 0.005 | 0.107 |
| **Swamp forest** | 0.693 | 0.918 |  | 0.098 | 0.030 | 0.002 |
| **Mangrove** | 0.908 | 0.816 | 0.871 |  | 0.063 | 0.263 |
| **Freshwater swamp** | 0.953 | 0.889 | 0.928 | 0.876 |  | 0.098 |
| **Coastal forest** | 0.905 | 0.695 | 0.936 | 0.648 | 0.748 |  |

**No Doubletons**

| **Overall Dissimilarity:** 0.944 **Overall** **Turnover:** 0.892 **Overall Nestedness:** 0.052 | | | | | | |
| --- | --- | --- | --- | --- | --- | --- |
|  | **Rainforest** | **Urban forest** | **Swamp forest** | **Mangrove** | **Freshwater swamp** | **Coastal forest** |
| **Rainforest** |  | 0.015 | 0.078 | 0.060 | 0.010 | 0.020 |
| **Urban forest** | 0.909 |  | 0.033 | 0.099 | 0.004 | 0.112 |
| **Swamp forest** | 0.685 | 0.915 |  | 0.102 | 0.032 | 0.003 |
| **Mangrove** | 0.906 | 0.816 | 0.868 |  | 0.064 | 0.268 |
| **Freshwater swamp** | 0.952 | 0.889 | 0.926 | 0.875 |  | 0.101 |
| **Coastal forest** | 0.903 | 0.687 | 0.934 | 0.643 | 0.744 |  |

**No Species <5 Specimens**

| **Overall Dissimilarity:** 0.944 **Overall** **Turnover:** 0.891 **Overall Nestedness:** 0.054 | | | | | | |
| --- | --- | --- | --- | --- | --- | --- |
|  | **Rainforest** | **Urban forest** | **Swamp forest** | **Mangrove** | **Freshwater swamp** | **Coastal forest** |
| **Rainforest** |  | 0.017 | 0.081 | 0.063 | 0.011 | 0.020 |
| **Urban forest** | 0.905 |  | 0.037 | 0.099 | 0.004 | 0.118 |
| **Swamp forest** | 0.677 | 0.912 |  | 0.107 | 0.035 | 0.004 |
| **Mangrove** | 0.904 | 0.817 | 0.862 |  | 0.064 | 0.274 |
| **Freshwater swamp** | 0.950 | 0.889 | 0.922 | 0.875 |  | 0.103 |
| **Coastal forest** | 0.902 | 0.679 | 0.931 | 0.638 | 0.741 |  |

**No Species <10 Specimens**

| **Overall Dissimilarity:** 0.945 **Overall** **Turnover:** 0.888 **Overall Nestedness:** 0.057 | | | | | | |
| --- | --- | --- | --- | --- | --- | --- |
|  | **Rainforest** | **Urban forest** | **Swamp forest** | **Mangrove** | **Freshwater swamp** | **Coastal forest** |
| **Rainforest** |  | 0.023 | 0.082 | 0.069 | 0.014 | 0.018 |
| **Urban forest** | 0.897 |  | 0.041 | 0.099 | 0.003 | 0.128 |
| **Swamp forest** | 0.665 | 0.907 |  | 0.115 | 0.038 | 0.005 |
| **Mangrove** | 0.898 | 0.818 | 0.856 |  | 0.065 | 0.282 |
| **Freshwater swamp** | 0.944 | 0.890 | 0.917 | 0.876 |  | 0.105 |
| **Coastal forest** | 0.899 | 0.665 | 0.927 | 0.632 | 0.742 |  |

**Table S8.** Number of species of vascular plants for each sampling site in Singapore from checklist data.

| Sampling Site | Habitat | No. of Plant Species | Reference |
| --- | --- | --- | --- |
| Nee Soon freshwater swamp | Freshwater swamp forest | 1150 | Wong et al., 2013[81] |
| Bukit Timah Nature Reserve | Rainforest | 1250 | Ho et al., 2019[69] |
| Kent Ridge | Urban-edge/disturbed forest | 420 | Tan et al., 2019[116] |
| Pulau Ubin | Mangrove | 245 | Lee et al., 2003[75] |
| Sungei Buloh Wetland Reserve | Mangrove | 249 | Tan et al., 1997[76] |
| Pulau Semakau | Mangrove | 165 | Teo et al., 2011[77] |

**Table S9.** Number of species from each guild, site and habitat type.

| Guild | Rainforest | Swamp Forest | Urban Forest | Mangrove Forest | | | |
| --- | --- | --- | --- | --- | --- | --- | --- |
|  | **Bukit Timah** | **Nee Soon** | **Kent Ridge** | **Pulau Ubin** | **Sungei Buloh** | **Pulau Semakau (Old)** | **Pulau Semakau (New)** |
| Phytophages | 471 | 447 | 125 | 155 | 110 | 109 | 71 |
| Pollinators | 15 | 17 | 1 | 29 | 29 | 20 | 20 |
| Fungivores | 400 | 380 | 83 | 67 | 46 | 27 | 12 |
| Parasitoids | 164 | 78 | 133 | 83 | 71 | 61 | 38 |
| Predators | 153 | 135 | 83 | 219 | 136 | 167 | 117 |
| Haematophages | 33 | 49 | 0 | 65 | 57 | 40 | 24 |
| Detritivores | 74 | 68 | 17 | 90 | 81 | 58 | 43 |

**Table S10.** Species turnover ANOSIM analysis results indicate distinct communities in each habitat type for each ecological guild. Pairwise p-value outputs are displayed in the bottom-left of the pairwise matrix while the R-statistics are displayed at the top-right.

**Phytophages**

| **Overall P:** 0.001 **Overall R:** 0.588 | | | | |
| --- | --- | --- | --- | --- |
|  | **Rainforest** | **Urban forest** | **Swamp forest** | **Mangrove** |
| **Rainforest** |  | 1.000 | 0.706 | 0.721 |
| **Urban forest** | 0.001 |  | 1.000 | 0.468 |
| **Swamp forest** | 0.001 | 0.029 |  | 0.665 |
| **Mangrove** | 0.001 | 0.003 | 0.001 |  |

**Pollinators**

| **Overall P:** 0.001 **Overall R:** 0.836 | | | |  |
| --- | --- | --- | --- | --- |
|  | **Rainforest** | **Swamp forest** | **Mangrove** | |
| **Rainforest** |  | 0.387 | 0.915 | |
| **Swamp forest** | 0.127 |  | 0.853 | |
| **Mangrove** | 0.001 | 0.004 |  | |

**Fungivores**

| **Overall P:** 0.001 **Overall R:** 0.351 | | | | |
| --- | --- | --- | --- | --- |
|  | **Rainforest** | **Urban forest** | **Swamp forest** | **Mangrove** |
| **Rainforest** |  | 1.000 | 0.726 | 0.435 |
| **Urban forest** | 0.001 |  | 1.000 | 0.088 |
| **Swamp forest** | 0.001 | 0.029 |  | 0.432 |
| **Mangrove** | 0.001 | 0.206 | 0.001 |  |

**Parasitoids**

| **Overall P:** 0.001 **Overall R:** 0.758 | | | | |
| --- | --- | --- | --- | --- |
|  | **Rainforest** | **Urban forest** | **Swamp forest** | **Mangrove** |
| **Rainforest** |  | 0.962 | 0.925 | 0.793 |
| **Urban forest** | 0.001 |  | 1.000 | 0.736 |
| **Swamp forest** | 0.018 | 0.067 |  | 0.711 |
| **Mangrove** | 0.001 | 0.001 | 0.006 |  |

**Predators**

| **Overall P:** 0.001 **Overall R:** 0.906 | | | | |
| --- | --- | --- | --- | --- |
|  | **Rainforest** | **Urban forest** | **Swamp forest** | **Mangrove** |
| **Rainforest** |  | 1.000 | 0.414 | 0.954 |
| **Urban forest** | 0.001 |  | 1.000 | 0.916 |
| **Swamp forest** | 0.109 | 0.067 |  | 0.913 |
| **Mangrove** | 0.001 | 0.001 | 0.002 |  |

**Haematophages**

| **Overall P:** 0.001 **Overall R:** 0.905 | | | |  |
| --- | --- | --- | --- | --- |
|  | **Rainforest** | **Swamp forest** | **Mangrove** | |
| **Rainforest** |  | 0.435 | 0.957 | |
| **Swamp forest** | 0.139 |  | 0.791 | |
| **Mangrove** | 0.001 | 0.002 |  | |

**Detritivores**

| **Overall P:** 0.001 **Overall R:** 0.853 | | | | |
| --- | --- | --- | --- | --- |
|  | **Rainforest** | **Urban forest** | **Swamp forest** | **Mangrove** |
| **Rainforest** |  | 0.613 | 0.487 | 0.949 |
| **Urban forest** | 0.008 |  | 1.000 | 0.904 |
| **Swamp forest** | 0.056 | 0.100 |  | 0.614 |
| **Mangrove** | 0.001 | 0.001 | 0.002 |  |

**Table S11.** Species turnover SIMPER analysis results indicate distinct communities in each habitat type for each ecological guild.

**Phytophages**

|  | **Within habitat (%)** | **Between habitats (%)** | | | |
| --- | --- | --- | --- | --- | --- |
|  |  | **Rainforest** | **Urban forest** | **Swamp forest** | **Mangrove** |
| **Rainforest** | 30.46 |  |  |  |  |
| **Urban forest** | 27.72 | 4.93 |  |  |  |
| **Swamp forest** | 34.21 | 18.38 | 5.18 |  |  |
| **Mangrove** | 12.37 | 1.29 | 4.13 | 1.67 |  |

**Pollinators**

|  | **Within habitat (%)** | **Between habitats (%)** | | |
| --- | --- | --- | --- | --- |
|  |  | **Rainforest** | **Swamp forest** | **Mangrove** |
| **Rainforest** | 41.27 |  |  |  |
| **Swamp forest** | 48.30 | 28.15 |  |  |
| **Mangrove** | 26.01 | 0.88 | 3.57 |  |

**Fungivores**

|  | **Within habitat (%)** | **Between habitats (%)** | | | |
| --- | --- | --- | --- | --- | --- |
|  |  | **Rainforest** | **Urban forest** | **Swamp forest** | **Mangrove** |
| **Rainforest** | 32.01 |  |  |  |  |
| **Urban forest** | 31.71 | 4.48 |  |  |  |
| **Swamp forest** | 36.88 | 19.40 | 4.61 |  |  |
| **Mangrove** | 10.58 | 1.87 | 8.26 | 1.34 |  |

**Parasitoids**

|  | **Within habitat (%)** | **Between habitats (%)** | | | |
| --- | --- | --- | --- | --- | --- |
|  |  | **Rainforest** | **Urban forest** | **Swamp forest** | **Mangrove** |
| **Rainforest** | 27.47 |  |  |  |  |
| **Urban forest** | 10.13 | 3.18 |  |  |  |
| **Swamp forest** | 59.26 | 10.76 | 1.14 |  |  |
| **Mangrove** | 12.00 | 2.40 | 2.43 | 2.84 |  |

**Predators**

|  | **Within habitat (%)** | **Between habitats (%)** | | | |
| --- | --- | --- | --- | --- | --- |
|  |  | **Rainforest** | **Urban forest** | **Swamp forest** | **Mangrove** |
| **Rainforest** | 29.22 |  |  |  |  |
| **Urban forest** | 34.03 | 4.60 |  |  |  |
| **Swamp forest** | 64.88 | 19.72 | 3.62 |  |  |
| **Mangrove** | 22.78 | 0.28 | 1.35 | 1.20 |  |

**Haematophages**

|  | **Within habitat (%)** | **Between habitats (%)** | | |
| --- | --- | --- | --- | --- |
|  |  | **Rainforest** | **Swamp forest** | **Mangrove** |
| **Rainforest** | 18.86 |  |  |  |
| **Swamp forest** | 56.42 | 10.55 |  |  |
| **Mangrove** | 27.40 | 0.61 | 9.27 |  |

**Detritivores**

|  | **Within habitat (%)** | **Between habitats (%)** | | | |
| --- | --- | --- | --- | --- | --- |
|  |  | **Rainforest** | **Urban forest** | **Swamp forest** | **Mangrove** |
| **Rainforest** | 14.99 |  |  |  |  |
| **Urban forest** | 20.18 | 7.12 |  |  |  |
| **Swamp forest** | 52.77 | 9.49 | 1.67 |  |  |
| **Mangrove** | 18.87 | 0.37 | 1.33 | 6.91 |  |

**Table S12.** Number of specimens from Singapore, Hong Kong and Brunei, as well as the size of the randomized subsample from Singapore.

|  | No. of Specimens | | | |  |
| --- | --- | --- | --- | --- | --- |
| Taxon | **Singapore** | **Singapore (Rarefied)** | **Hong Kong** | **Brunei** | **Thailand** |
| Dolichopodidae | 17860 | 2800 | 2563 | 2798 | 924 |
| Phoridae | 2134 | 560 | 562 | 272 | - |
| Mycetophilidae | 223 | 180 | 186 | - | - |
| Total | **20217** | **3540** | **3311** | **3070** | **924** |

**Table S13.** Collection periods and trap localities; M = mangroves, SF = swamp forest, UF = urban forest, TF = tropical rainforest, CF = coastal forest, FS = freshwater swamp. Numbers in brackets indicate traps with <100 specimens in total that were excluded from the diversity analyses.

| Sampling period | Location | Habitat type | GPS coordinates | No. of traps | Total no. of weekly samples | Used for guild-level analyses |
| --- | --- | --- | --- | --- | --- | --- |
| Singapore | | | | | | |
| Apr 2012 – Mar 2014 | Pulau Ubin | M | 1°24'36.3"N 103°59'25.5"E | 3 | 72 | Y |
|  | Pulau Semakau original | M | 1°12'17.6"N 103°45'37.7"E | 3 | 72 | Y |
|  | Pulau Semakau replanted | M | 1°12'03.1"N 103°45'45.4"E | 3 | 72 | Y |
|  | Sungei Buloh Wetland Reserve | M | 1°26'46.3"N 103°43'49.9"E | 2 | 48 | Y |
|  | Nee Soon freshwater swamp | SF | 1°23'00.3"N 103°48'46.5"E | 2 | 48 | Y |
| May 2014 – Jun 2014 | Mandai Nature Park | M | 1°26'18.3"N 103°45'49.7"E | 3 (1) | 6 | N |
|  | Pulau Tekong | M | 1°25'47.3"N 104°03'46.3"E | 3 (2) | 6 | N |
|  | Sarimbun | M | 1°25'59.1"N 103°41'21.8"E | 3 (1) | 6 | N |
| Nov 2014 – May 2015 | Nee Soon freshwater swamp | SF | 1°23'00.3"N 103°48'46.5"E | 2 | 14 | Y |
| Apr 2015 – Sep 2015 | NUS | UF | 1°17'49.6"N 103°46'35.7"E | 4 | 24 | Y |
| Mar 2016 – Aug 2016 | Pulau Ubin | M | 1°25'11.64"N 103°56'6.25"E | 10 | 60 | Y |
|  | Sungei Buloh Wetland Reserve | M | 1°26'43.20"N 103°43'5.10"E | 10 | 60 | Y |
|  | Labrador Park | M | 1°16'13.3"N 103°48'10.1"E | 3 | 18 | N |
|  | Labrador Park | CF | 1°16'05.4"N 103°48'16.2"E | 2 | 18 | N |
| Aug 2016 – Oct 2017 | Bukit Timah Nature Reserve primary forest | TF | 1°21'13.90"N 103°46'47.57"E | 3 | 45 | Y |
|  | Bukit Timah Nature Reserve old secondary forest | TF | 1°21'17.96"N 103°46'54.01"E | 3 | 45 | Y |
|  | Bukit Timah Nature Reserve maturing secondary forest | TF | 1°21'4.57"N 103°46'53.80"E | 3 | 45 | Y |
| Apr 2017 – 20 Sep 2017 | NUS | UF | 1°17'49.6"N 103°46'35.7"E | 4 | 18 | N |
| Sep 2017 – Dec 2017 | NUS | UF | 1°17'45.3"N 103°46'13.8"E | 3 (1) | 16 | N |
| Mar 2018 – Jun 2018 | Bishan-Ang Moh Kio Park | UF | 1°21'35.7"N 103°50'49.9"E | 2 | 26 | N |
|  | Enabling Village | UF | 1°17'13.6"N 103°48'53.3"E | 1 | 13 | N |
|  | Esplanade Theatre | UF | 1°17'26.4"N 103°51'17.9"E | 1 | 13 | N |
|  | Sungei Buloh Wetland Reserve | M | 1°26'52.45"N 103°43'24.16"E | 4 (1) | 16 | N |
|  | Kranji Marshes | FS | 1°25'0.56"N 103°43'43.50"E | 3 | 12 | N |
|  | Lim Chu Kang | M | 1°26'48.80"N 103°42'35.71"E | 2 | 8 | N |
|  | Mandai Nature Park | M | 1°26'37.96"N 103°45'59.70"E | 4 | 16 | N |
|  | Pulau Ubin | CF | 1°24'26.3"N 103°57'16.3"E | 3 | 12 | N |
|  | Pulau Ubin | M | 1°24'32.2"N 103°57'12.1"E | 8 | 32 | N |
|  | Labrador Park | M | 1°16'13.3"N 103°48'10.1"E | 5 | 20 | N |
|  | Labrador Park | CF | 1°16'05.4"N 103°48'16.2"E | 4 (2) | 20 | N |
| Mar 2019 – Jun 2019 | Coney Island | M | 1°24'37.3"N 103°55'23.1"E | 5 (2) | 15 | N |
|  | Kranji Marshes | FS | 1°25'11.0"N 103°43'54.3"E | 4 | 15 | N |
|  | Pulau Ubin | CF | 1°25'34.7"N 103°56'29.2"E | 1 | 15 | N |
|  | Pulau Ubin | M | 1°25'05.3"N 103°56'06.5"E | 7 (6) | 15 | N |
| Hong Kong | | | | | | |
| Nov 2017 – Dec 2017,  May 2018 – Jul 2018 | Ha Pak Nai | M | 22°25'31.48"N 113°56'20.11"E | 6 | 30 | Y |
|  | Hang Mei | M | 22°15'9.83"N 113°52'5.84"E | 5 | 25 | Y |
|  | Ho Chung | M | 22°21'13.18"N 114°15'7.45"E | 6 | 30 | Y |
|  | Lai Chi Wo | M | 22°31'37.63"N 114°15'43.63"E | 5 | 25 | Y |
|  | Nam Chung | M | 22°31'31.62"N 114°12'28.94"E | 5 | 25 | Y |
|  | Sai Keng | M | 22°25'13.48"N 114°16'4.66"E | 5 | 25 | Y |
|  | Sam A Chung | M | 22°30'29.84"N 114°16'20.93"E | 5 | 25 | Y |
|  | Sam A Tsuen | M | 22°30'55.22"N 114°16'16.36"E | 5 | 25 | Y |
|  | Sha Tau Kok | M | 22°32'4.34"N 114°12'39.78"E | 10 | 50 | Y |
|  | Sheung Pak Nai | M | 22°27'7.09"N 113°57'45.11"E | 5 | 25 | Y |
|  | Shui Hau | M | 22°13'9.70"N 113°55'8.33"E | 5 | 25 | Y |
|  | So Lo Pun | M | 22°32'17.20"N 114°15'21.49"E | 5 | 25 | Y |
|  | Tai O | M | 22°15'28.44"N 113°51'48.96"E | 6 | 30 | Y |
|  | Tai Tam | M | 22°14'46.10"N 114°13'24.02"E | 3 | 15 | Y |
|  | Tai Tan | M | 22°26'18.85"N 114°19'59.77"E | 1 | 5 | Y |
|  | To Kwa Peng | M | 22°25'43.07"N 114°19'59.30"E | 5 | 25 | Y |
|  | Tsim Bei Tsui | M | 22°29'20.47"N 113°59'53.95"E | 5 | 25 | Y |
|  | Tung Chung | M | 22°16'52.50"N 113°55'44.04"E | 6 | 30 | Y |
|  | Wong Chuk Wan | M | 22°23'44.27"N 114°17'10.21"E | 5 | 25 | Y |
|  | Yim Tin Tsai | M | 22°22'32.74"N 114°18'5.76"E | 5 | 25 | Y |
| Brunei | | | | | | |
| Jul 2014 – Nov 2014 | Pulau Berambang | M | 4°54'7.44"N 115°1'17.94"E | 2 | 10 | Y |
|  | Labu Forest Reserve | M | 4°51'41.75"N 115°6'59.69"E | 2 | 10 | Y |
|  | Tutong Forest | M | 4°46'9.54"N 114°36'20.64"E | 2 | 10 | Y |
