# Supplementary material for: Mangroves are an overlooked hotspot of insect diversity despite low plant diversity
Source: BMC Biol. 2021 Sep 14;19:202. doi: 10.1186/s12915-021-01088-z (PMC8442405; doi:10.1186/s12915-021-01088-z)
Supplement: Supplementary file 2 — Additional file 2: Figure S1. Sampling locations in the Oriental realm and in Singapore. Figure S2. Arthropod orders sampled in this study and their species proportions. Figure S3. Insect alpha-diversity across tropical forest habitats. Figure S4. Breakaway and CNE species estimates for each habitat. Figure S5. Insect alpha-diversity across tropical forest habitats for the core dataset. Figure S6. Insect species turnover from the core dataset. Figure S7. Species diversity across habitats split by ecological guild. Figure S8. Species diversity across habitats (mangroves split) split by ecological guild. Figure S9. Species diversity and turnover from Singapore, Brunei, and Hong Kong datasets. [file 12915_2021_1088_MOESM2_ESM.docx]

**
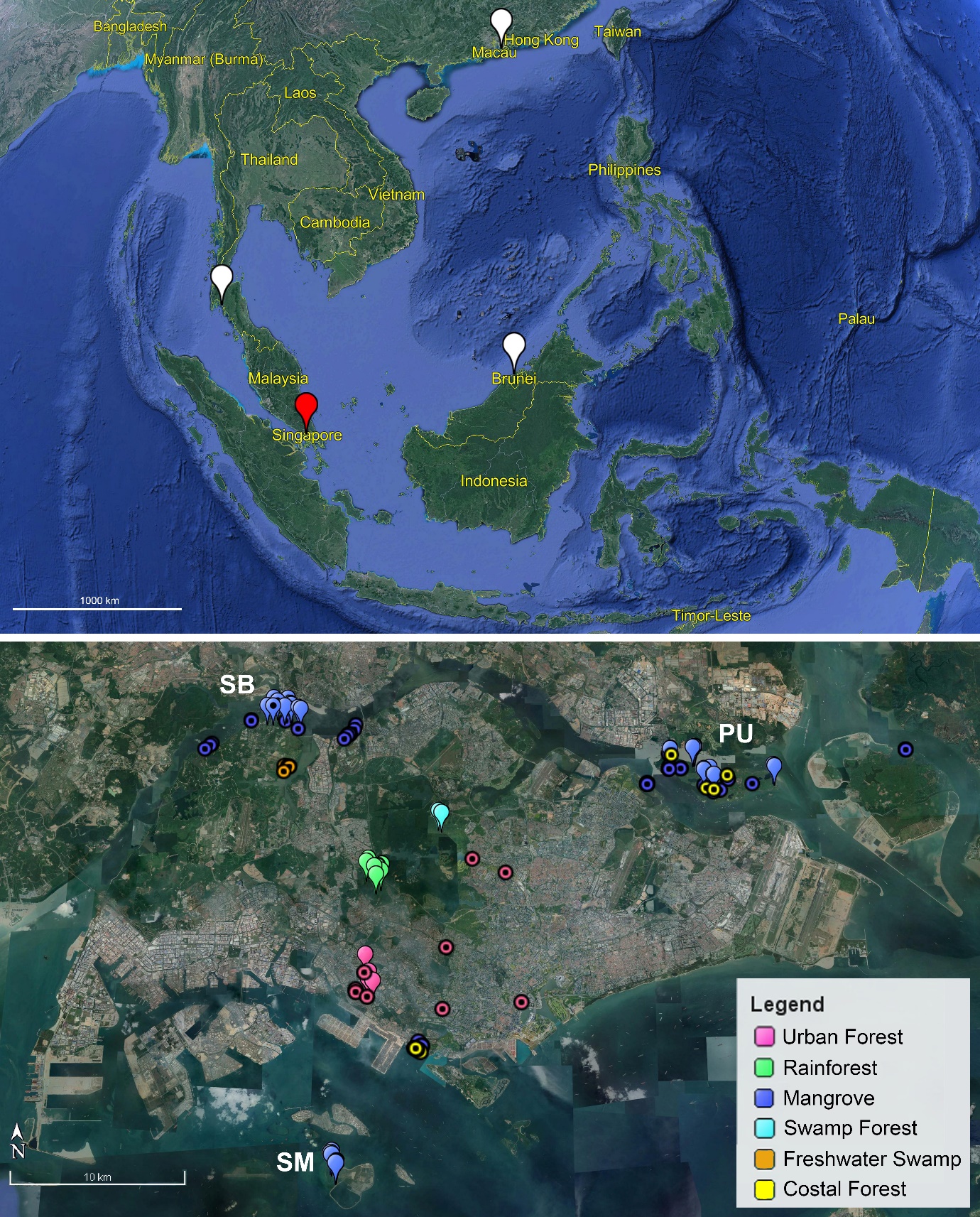
**

**Figure S1.** Sampling localities in the Oriental Realm (*top*: Singapore, red; other countries, white) and within Singapore (*bottom*: circular markers indicate trapping sites excluded from the species turnover analyses; pin markers with dot indicate traps excluded from guild-level analyses).

**Figure S2.** Arthropod orders sampled with Malaise traps in this study and their species proportions. The number beside each order indicates the number of species sampled based on 3% p-distance objective clustering.


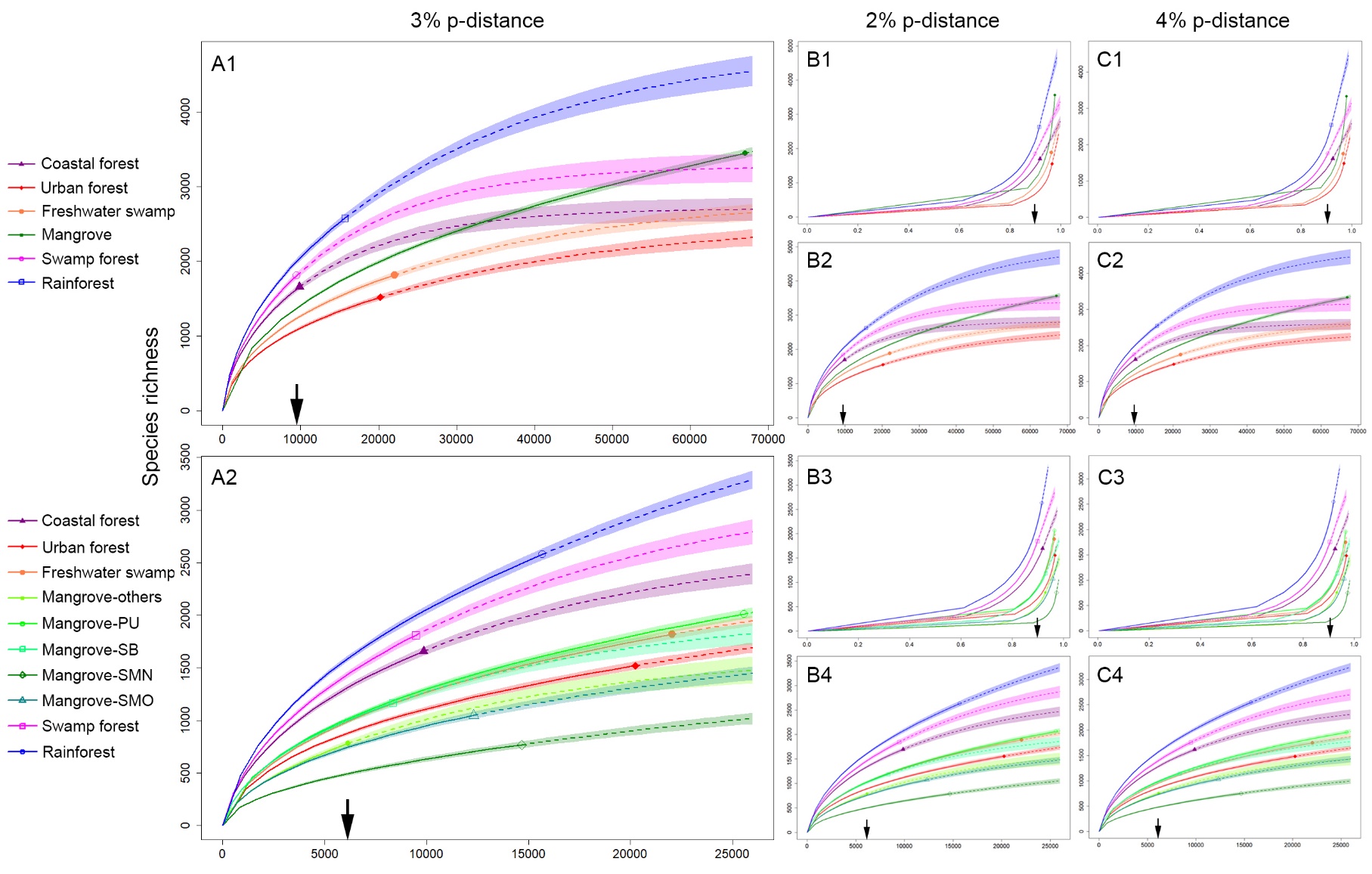


**Figure S3.** Insect alpha-diversity across tropical forest habitats rarefied by specimens (A1 & 2, B2 & 4, C2 & 4) and coverage (B1 & 3, C1 & 3), for 2% (B1 – 4), 3% (A1 – 2) and 4% (C1 – 4) p-distances mOTUs. Mangroves are treated as a single habitat (top) and split by site in a separate analysis (bottom): Pulau Ubin (PU), Sungei Buloh (SB), Pulau Semakau old grove (SMO), Pulau Semakau new grove (SMN); solid lines = rarefaction; dotted = extrapolations. The arrow on the x-axis indicate the point of rarefaction at which species richness comparisons were made.


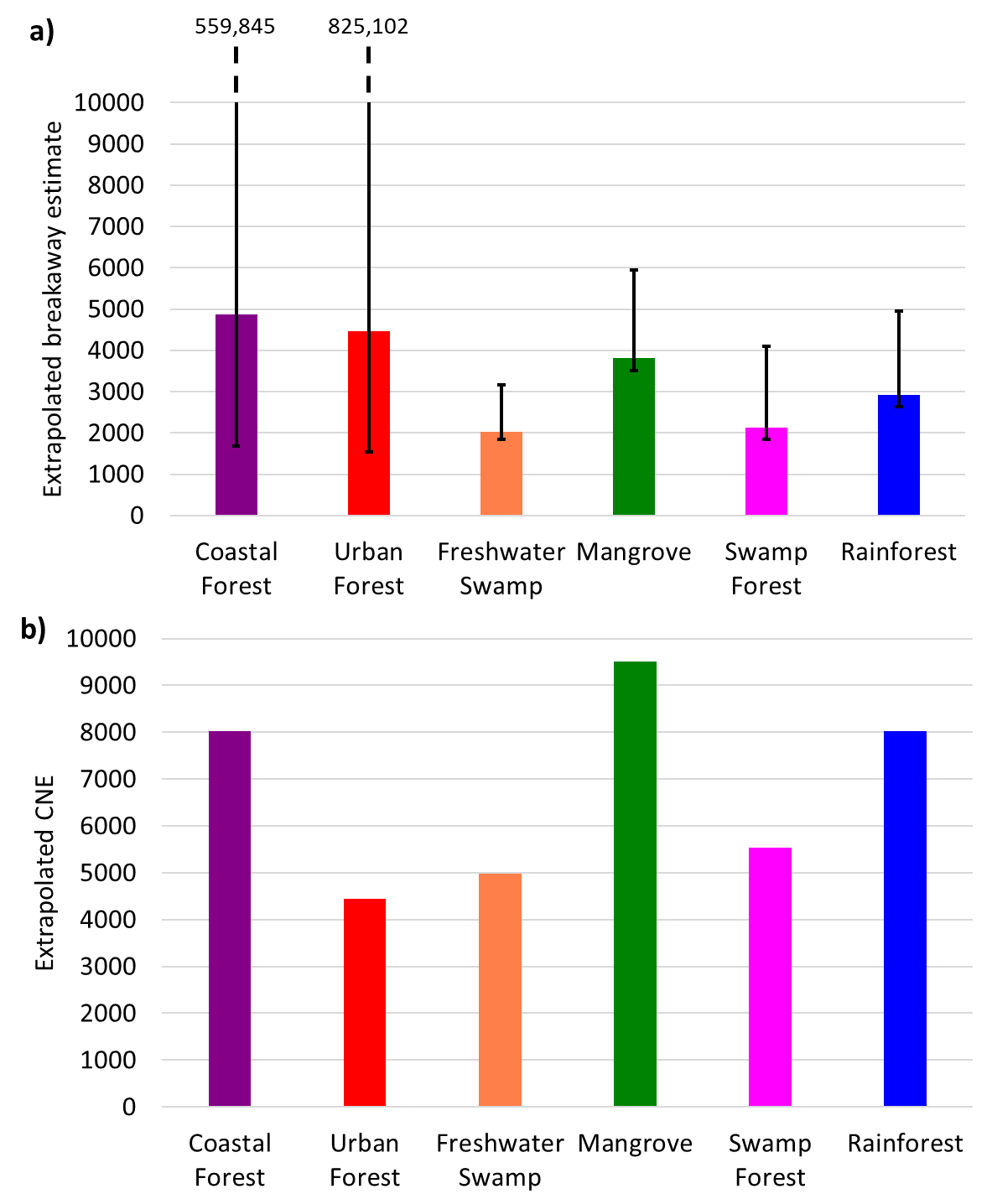


**Figure S4.** Species estimates for each habitat based on a) breakaway estimates and b) CNE extrapolation find mangroves more species-rich than swamp forest and rainforest habitats.


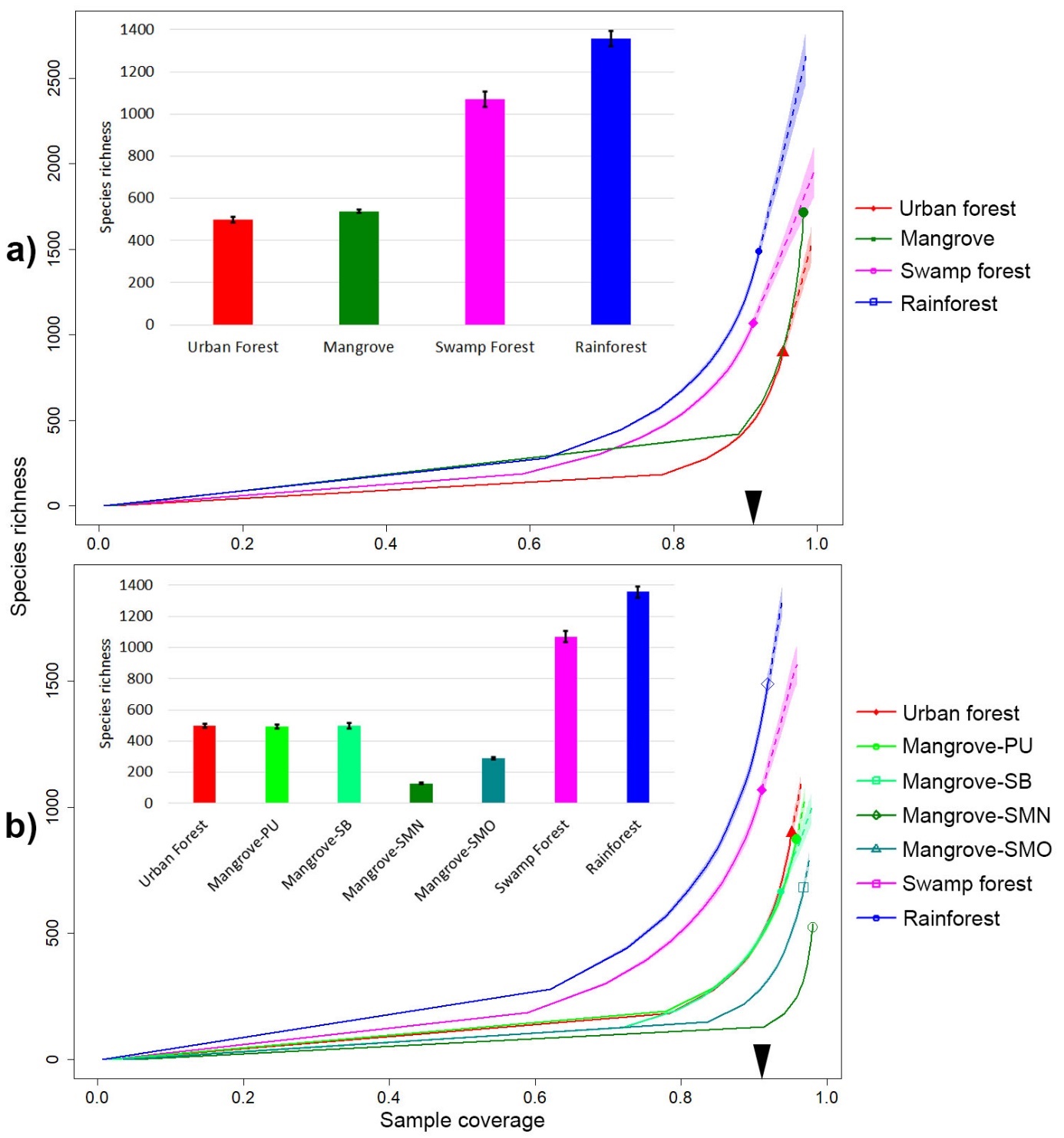


**Figure S5.** Insect alpha-diversity across tropical forest habitats for the core dataset. (a) Mangroves treated as one habitat; (b) Comparison of mangrove sites: Pulau Ubin (PU), Sungei Buloh (SB), Pulau Semakau old-growth (SMO), Pulau Semakau new-growth (SMN); solid lines = rarefaction; dotted = extrapolations. The arrow on the x-axis indicates the point of rarefaction where species richness comparisons were made, which is reflected in the bar charts with associated 95% confidence intervals.


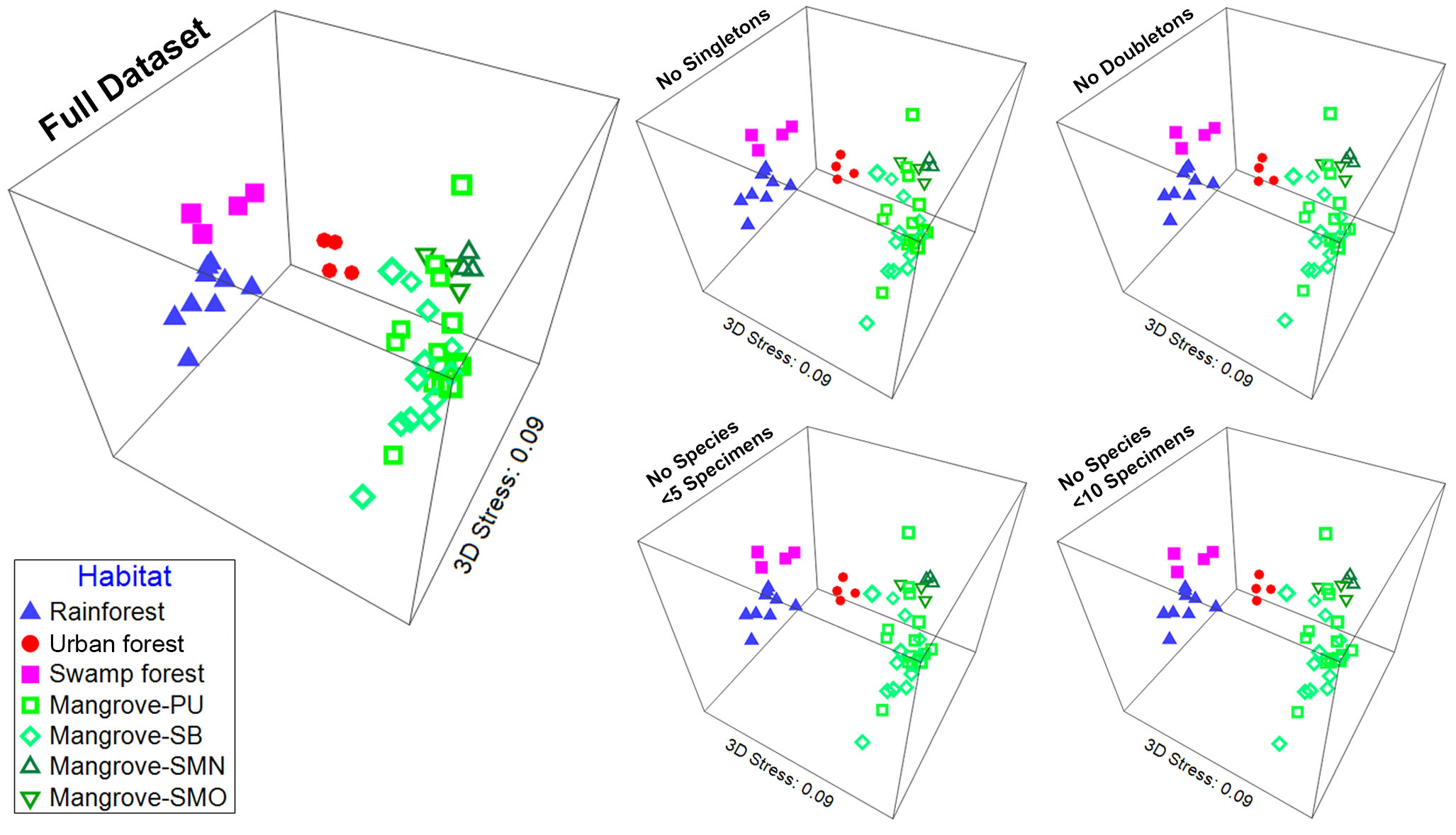


**Figure S6.** Insect communities from the core dataset are distinct across tropical forest habitats based on Bray-Curtis distances. as illustrated with 3D NMDS plots, regardless of whether rare species are removed.

**
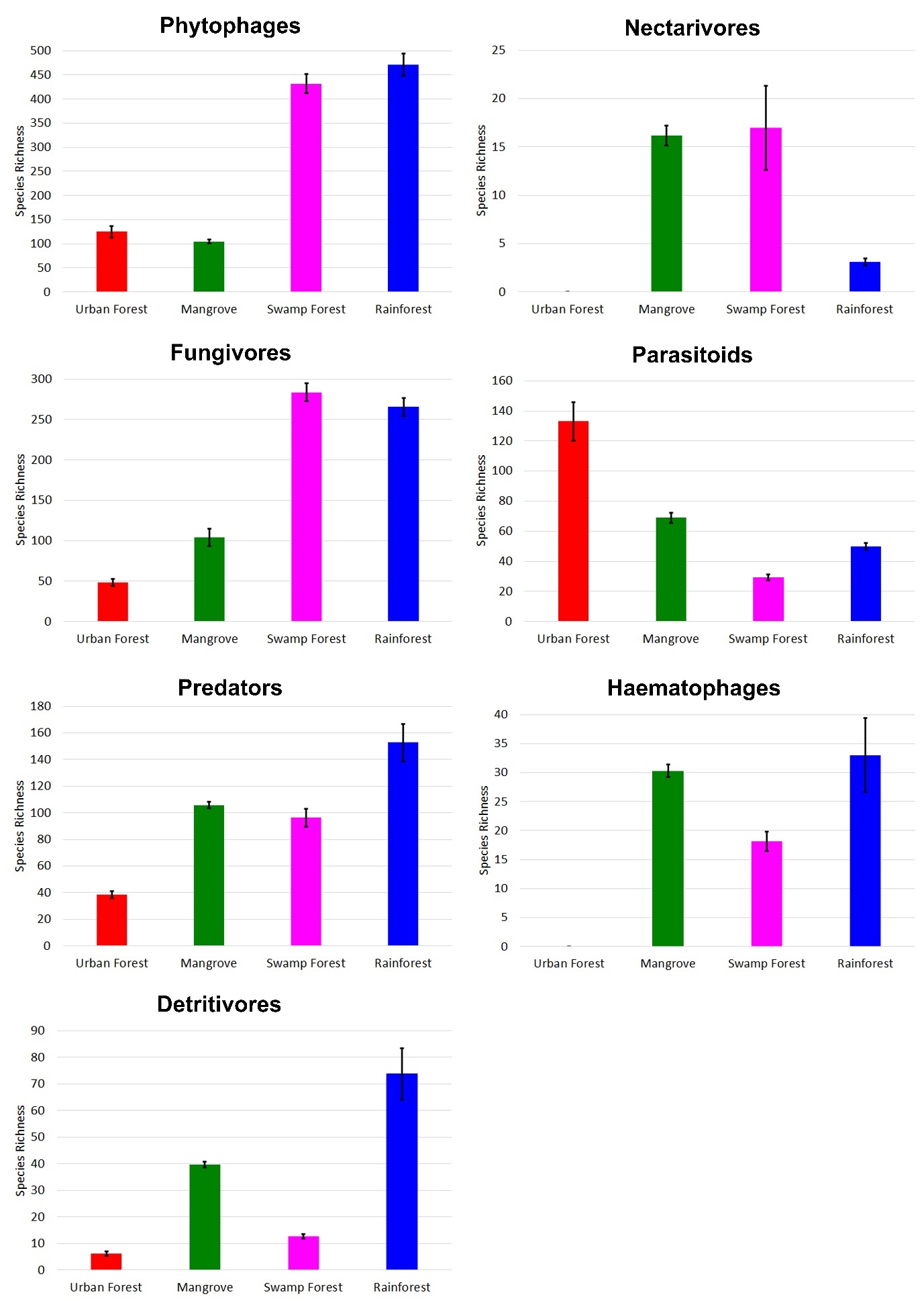
**

**Figure S7.** Comparison of species diversity across habitats (3% p-distance mOTUs) split by ecological guild. Values were taken at the point of rarefaction based on lowest coverage and include 95% confidence intervals. Mangrove sites are represented as a single habitat type.


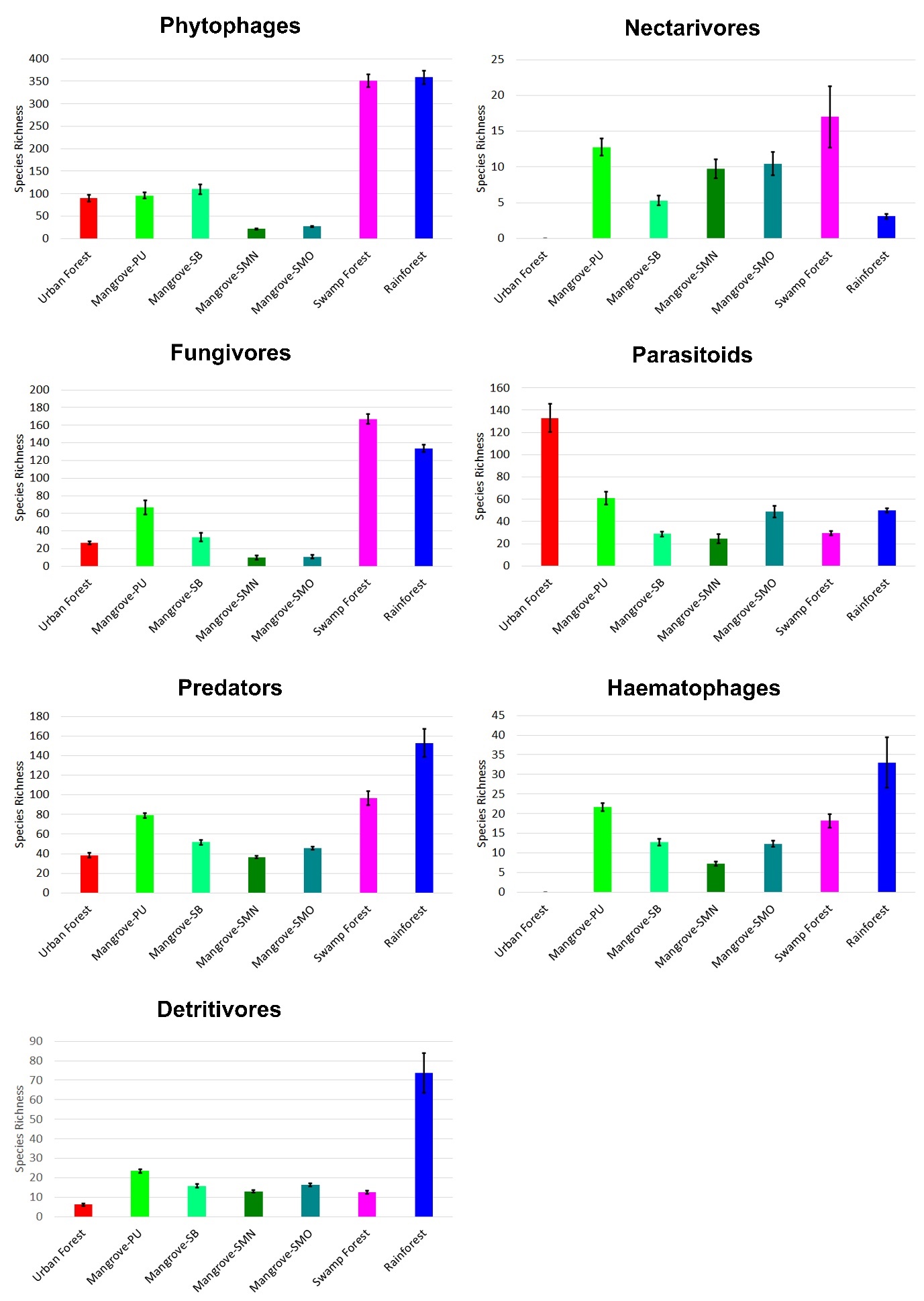


**Figure S8.** Comparison of species diversity across habitats (3% p-distance mOTUs) split by ecological guild. Values were taken at the point of rarefaction based on lowest coverage and include 95% confidence intervals. Mangrove sites are represented by Pulau Ubin (PU), Sungei Buloh (SB), Pulau Semakau old grove (SMO), Pulau Semakau new grove (SMN).

**Figure S9.** High species diversity and turnover for mangroves from Singapore, Brunei, and Hong Kong based on three Diptera families. Singapore data are rarefied to specimen numbers from Brunei and HK (error bars = 95% confidence intervals).
